# Supplementary material for: Electronic Health Record Skills Workshop for Medical Students
Source: MedEdPORTAL. 2019 Oct 25;15:10849. doi: 10.15766/mep_2374-8265.10849 (PMC6946580; doi:10.15766/mep_2374-8265.10849)
Supplement: Supplementary file 1 — A. Case 1.docx B. Case 2.docx C. Case 3.docx D. Student Guide.docx E. Facilitator Guide.docx F. Pretest and Posttest.docx G. EHR Presentation.pptx H. PDQI-9.pdf [file mep-15-10849-s001.zip › _Educational_Summary_Report_10849.pdf]

MedEdPORTAL® The AAMC Journal of Teaching and Learning Resources

Original Publication Open Access

Electronic Health Record Skills Workshop for Medical Students

Jillian Zavodnick, MD*, Tasha Kouvatsos, MD

*Corresponding author: jillian.zavodnick@jefferson.edu

Abstract

Introduction: The adoption of electronic health records (EHRs) presents new challenges for information gathering, documentation, and patient care. Medical students spend a significant amount of time using the EHR during their clerkship experiences and will continue to do so as they progress to residency. However, formal training varies between institutions and leaves gaps in data-gathering skills, documentation skills, and order entry—these three skills formed the basis for our learning objectives. We designed a workshop using a simulated EHR to teach these skills. **Methods:** We offered the workshop during a class-wide transition-to-internship course for senior medical students. After a brief didactic, students worked in small groups using a simulated EHR to complete cases addressing each of the three learning objectives. Faculty facilitators assisted groups and then guided a large-group discussion of the challenges encountered during the cases. **Results:** Twenty-five senior medical students attended the first optional workshop. Of these students, 66.7% recommended that the workshop continue to be included in the curriculum. Comments from those who recommended otherwise suggested that many of them would recommend the workshop if it used our local EHR (Epic). Correct answers to the factual questions increased for most questions between the pretest and the posttest. Confidence to perform all skills targeted in the learning objectives increased between the pretest and the posttest. **Discussion:** This EHR workshop was well received by senior medical students and increased confidence in EHR skills, including data gathering, documentation, and handling unsolicited information with a plan including order entry.

Keywords
Electronic Health Records, Electronic Medical Record, EHR, EMR, Electronic Note, Transition to Internship, Informatics/Health IT

Educational Objectives

By the end of this activity, learners will be able to:

1. Query the electronic health record (EHR) in a complete yet concise way to obtain patient care information.
2. Create safe, high-quality electronic documentation, remaining aware of EHR pitfalls and the multiple purposes of the medical note.
3. Implement management plans for information obtained from the EHR, incorporating computerized physician order entry and patient communication when necessary.

Introduction

The adoption of electronic health records (EHRs) presents new challenges for information gathering, documentation, and patient care. Medical students spend a significant amount of time using the EHR during their clerkship experiences and will continue to do so as they progress to residency.[1,2] However, formal training varies between institutions and leaves gaps in data-gathering skills,[3] documentation skills,[4] and order entry.[5] We designed a workshop using a simulated EHR to teach these skills.

Citation:
Zavodnick J, Kouvatsos T. Electronic health record skills workshop for medical students. *MedEdPORTAL*. 2019;15:10849.
https://doi.org/10.15766/mep_2374-8265.10849

Although medical schools train students to ask the most useful questions during patient interviews when they teach history-taking skills, similar strategies for obtaining the most useful data from the EHR are not routinely taught. Routine EHR training and a year of clinical experience did not prevent 79% of medical students from overlooking documentation of a previous myocardial infarction in a standardized patient presenting with acute chest pain.[3] Documentation in the EHR, by students and others, is rife with copy-and-paste text, a process that decreases documentation quality and interferes with diagnostic reasoning.[4,6] Students value writing orders for their patients, but this becomes less common with the adoption of computerized physician order entry,[5] even as the Alliance for Clinical Education recommends that this activity be included in clerkship education

Copyright © 2019 Zavodnick and Kouvatsos. This is an open-access publication distributed under the terms of the Creative Commons Attribution license.

and the Alliance for Academic Internal Medicine identifies order entry as a core entrustable professional activity for entering residency.[7,8] These gaps in EHR skills informed our workshop objectives.

Our workshop was targeted toward senior medical students and offered during a transition-to-internship course. The cases included medical, surgical, and pediatric patients in an attempt to be relevant to as many future specialties as possible. However, the material is at a level appropriate for any medical student with some clinical experience.

Previously described EHR workshops focus predominantly on communication skills surrounding EHR use during patient encounters.[9,10] A workshop using a simulated EHR to teach ICD-10 codes has also been published.[11] To our knowledge, ours is the first disseminated workshop of its kind with a focus on information-gathering, documentation, and direct patient care skills.

Methods

We offered the workshop during a class-wide transition-to-internship course. All students at our institution had access to the Regenstrief Institute's Teaching EMR (tEMR), a simulated EHR available to several institutions. It consists of real, misidentified patient records that can be customized to need by working with tEMR staff. For institutions without this resource, we recommend working with the information technology department to see if case data can be loaded in a training environment of the local EHR (e.g., the Epic Playground); discuss the schedule for deleting this information, as it may be necessary to input the data on the day of the workshop. Case 1 (Appendix A) consisted of notes and imaging reports; case 2 (Appendix B) consisted of an admission note, a progress note, and laboratory results; and case 3 (Appendix C) consisted of laboratory results and a telephone encounter note. All cases included additional history given in the Student Guide (Appendix D).

The workshop lasted 90 minutes, and students were asked to bring an internet-capable laptop or tablet. Wireless internet access was available. Two faculty members facilitated using the Facilitator Guide (Appendix E), and a computer and projector were used. We started with a pretest (Appendix F), delivered a brief slide presentation (Appendix G; the presentation referred to the Physician Documentation Quality Instrument, included here in Appendix H), and then distributed the Student Guide (Appendix D) to each student. We divided into groups of four students each and assigned case 1, 2, or 3 to each group. Groups were given 20 minutes to work through their assigned case, and then we reconvened as a large group. For each case, the groups who completed it described their approach and the challenges they encountered. The facilitators used the teaching points in the Facilitator Guide to guide the discussion, both among the individual groups as they tackled their cases and during the large-group discussion. A brief wrap-up summarized the learning points, and an identical posttest (Appendix F) was completed to evaluate changes in knowledge and confidence. The confidence questions were specifically tailored to the learning objectives.

Results

Twenty-five senior medical students attended the first optional workshop, which was facilitated by both authors. Of the students, 66.7% recommended that the workshop continue to be included in the curriculum. Comments from those who recommended otherwise suggested that many of them would recommend the workshop if it had used our local EHR (Epic).

Correct answers to the factual questions increased for most questions between the pretest and the posttest (Figure 1). These questions touched on all three learning objectives, with the first three questions addressing the multiple purposes of the medical note and EHR pitfalls (second objective), question 4 addressing the approach to EHR queries (first objective), and question 5 addressing the approach to responding to EHR data (third objective). Confidence to perform all skills targeted in the learning objectives increased between the pretest and the posttest (Figure 2). Learners were asked about their confidence in querying the EHR (first objective), creating documentation (second objective), and initiating management plans (third objective).

Discussion

This EHR workshop was well received by senior medical students and increased confidence in EHR skills including data gathering, documentation, and handling unsolicited information with a plan including order entry. Confidence in these skills was very low prior to the session, and attendance was better than we anticipated for a fully voluntary session, implying that students recognized these gaps in their skills. To increase confidence above the posttest levels prior to the start of residency, it is our hope that students will take the concepts and approaches they learned and put them into practice during their final months of medical school. Programs with more time to explore this topic could consider extending the time of the workshop so that each group can work through each task, developing additional tasks and expanding the workshop into a series, or providing additional tasks as take-home cases.

Copyright © 2019 Zavodnick and Kouvatsos. This is an open-access publication distributed under the terms of the Creative Commons Attribution license.

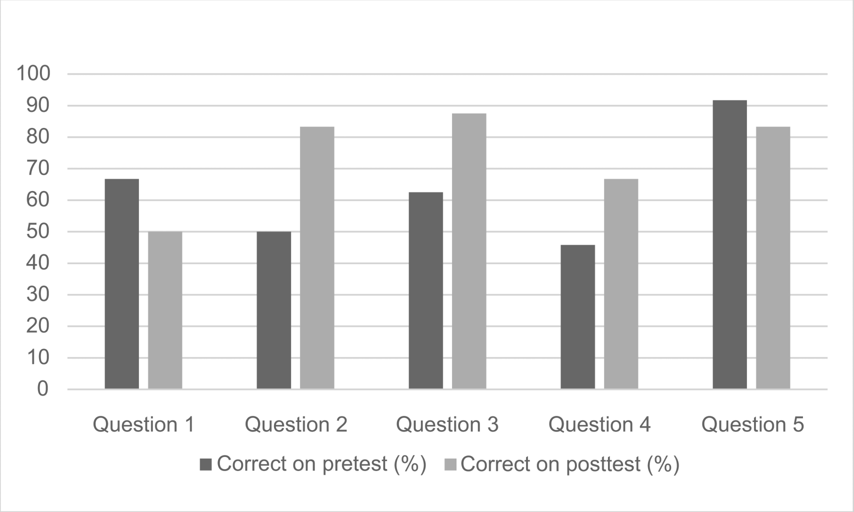

Figure 1. Percentage of students (*n* = 24) who correctly answered knowledge questions pretest and posttest.

Preparation for this workshop began several months in advance, with the development of the cases in conjunction with tEMR staff. Altering patient records to best reflect the teaching points of each case and playing through the case in advance to uncover inconsistencies were the most time-consuming parts of the preparation. We advise early development or trialing of the cases, regardless of the EHR platform. Some materials refer to dates of EHR data (e.g., the date of a note or laboratory report). These may vary in development of cases at another institution, regardless of platform, and are included as examples.

Some students were surprised that the workshop focused on an overall cognitive approach to EHR use rather than a list of EHR shortcuts such as how to make a smartphrase or use an orderset; some of these students were pleased with the workshop, but some would have preferred a more how-to approach. Educators planning a voluntary activity should clearly advertise the learning objectives. We also recommend careful attention to the pretest and posttest knowledge questions; ours left some room for ambiguity, which sparked a valuable discussion but impeded the accurate assessment of knowledge gained during the session. We found our assessment useful for evaluating confidence in the

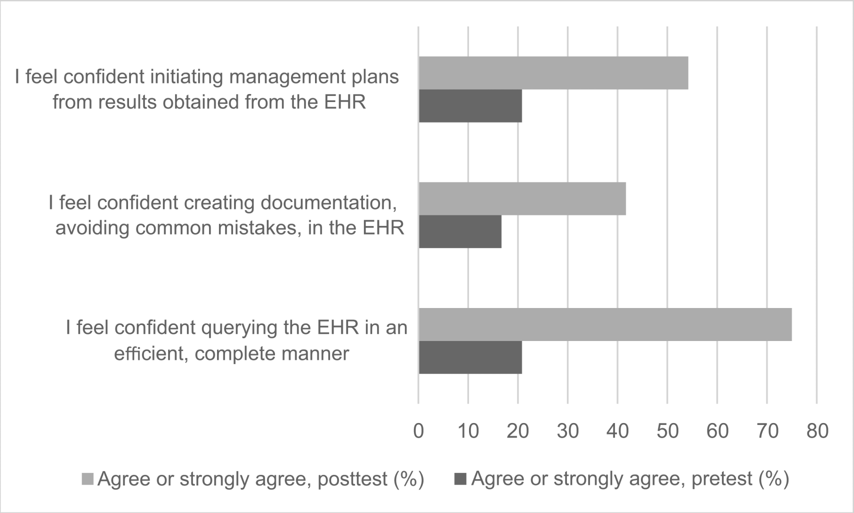

Figure 2. Percentage of residents (*n* = 24) who selected “agree” or “strongly agree” in response to questions about confidence in performing learning objectives pretest and posttest. EHR, electronic health record.

Copyright © 2019 Zavodnick and Kouvatsos. This is an open-access publication distributed under the terms of the Creative Commons Attribution license.

skills that were our primary learning objectives but would like to know more about the impact of this workshop on knowledge than we were able to gain from our questions.

We received valuable narrative feedback from the students that will influence next year's workshop; for example, we will explore using the Epic Playground as our platform while reminding students that their future training site may use a different EHR and that the workshop aims to teach generalizable, transferrable skills analogous to history-taking skills. Institutions adapting our curriculum can use their own EHR training environment, as students placed a premium on learning these skills within a system with which they were already familiar, and this workshop can be easily adapted to Epic or other systems. We also received requests to provide additional examples of documentation pitfalls and additional order entry practice. Ultimately, this material will be incorporated into a longitudinal fourth-year curriculum and will likely be broken down into individual topics with an opportunity for expansion of each topic, as well as incorporation of new topics such as controlled substance database use and panel management skills.

This workshop was found valuable by students and addresses gaps that have been demonstrated both in the literature and in our students' presession self-reported confidence. As EHRs become an ever more integral part of medical practice, their role in medical care and the skills to use them properly should become as much a part of the medical school curriculum as the proper use of a stethoscope.

Appendices

A. Case 1.docx

B. Case 2.docx

C. Case 3.docx

D. Student Guide.docx

E. Facilitator Guide.docx

F. Pretest and Posttest.docx

G. EHR Presentation.pptx

H. PDQI-9.pdf

All appendices are peer reviewed as integral parts of the Original Publication.

Jillian Zavodnick, MD: Clinical Assistant Professor, Department of Medicine, Sidney Kimmel Medical College at Thomas Jefferson University

Tasha Kouvatsos, MD: Clinical Assistant Professor, Department of Medicine, Sidney Kimmel Medical College at Thomas Jefferson University

Acknowledgments
The authors wish to acknowledge Jeff Warvel of the Regenstrief Institute and Anthony J. Frisby, PhD, Director of Jefferson University Libraries, for their assistance in accessing and customizing the training electronic health record.

Disclosures
None to report.

Funding/Support
None to report.

Prior Presentation
Zavodnick J, Kouvatsos T. 21st century clinical skills: teaching electronic health record skills in a transition-to-internship course. Poster presented at: Academic Internal Medicine Week; April 15, 2019; Philadelphia, PA.

Ethical Approval
The Thomas Jefferson University Institutional Review Board approved this study.

References

1. Chi J, Kugler J, Chu IM, et al. Medical students and the electronic health record: "an epic use of time." *Am J Med*. 2014;127(9):891-895. https://doi.org/10.1016/j.amjmed.2014.05.027
2. Chen L, Guo U, Illipparambil LC, et al. Racing against the clock: internal medicine residents' time spent on electronic health records. *J Grad Med Educ*. 2016;8(1):39-44. https://doi.org/10.4300/JGME-D-15-00240.1
3. Yudkowsky R, Galanter W, Jackson R. Students overlook information in the electronic health record. *Med Educ*. 2010;44(11):1132-1133. https://doi.org/10.1111/j.1365-2923.2010.03838.x
4. Heiman HL, Rasminsky S, Bierman JA, et al. Medical students' observations, practices, and attitudes regarding electronic health record documentation. *Teach Learn Med*. 2014;26(1):49-55. https://doi.org/10.1080/10401334.2013.857337
5. Knight AM, Kravet SJ, Harper GM, Leff B. The effect of computerized provider order entry on medical student clerkship experiences. *J Am Med Inform Assoc*. 2005;12(5):554-560. https://doi.org/10.1197/jamia.M1839
6. Hartzband P, Groopman J. Off the record—avoiding the pitfalls of going electronic. *N Engl J Med*. 2008;358(16):1656-1658. https://doi.org/10.1056/NEJMp0802221
7. Elnicki DM, Aiyer MK, Cannarozzi ML, et al. An entrustable professional activity (EPA)-based framework to prepare fourth-year medical students for internal medicine careers. *J Gen*

Copyright © 2019 Zavodnick and Kouvatsos. This is an open-access publication distributed under the terms of the Creative Commons Attribution license.

Intern Med. 2017;32(11):1255-1260. https://doi.org/10.1007/s11606-017-4089-8

8. Hammoud MM, Dalrymple JL, Christner JG, et al. Medical student documentation in electronic health records: a collaborative statement from the Alliance for Clinical Education. *Teach Learn Med*. 2012;24(3):257-266. https://doi.org/10.1080/10401334.2012.692284
9. Alkureishi M, Lee WW, Farnan J, Arora V. Breaking away from the iPatient to care for the real patient: implementing a patient-centered EMR use curriculum. *MedEdPORTAL*. 2014;10:9953. https://doi.org/10.15766/mep_2374-8265.9953
10. Han H, Waters T, Lopp L. Preserving patient relationship-centered care while utilizing EHRs. *MedEdPORTAL*. 2014;10:9729. https://doi.org/10.15766/mep_2374-8265.9729
11. Lee EL, Chisty A, Williams P, Brady W. An innovative curriculum using a simulated electronic health record to teach internal medicine residents about ICD-10-CM. *MedEdPORTAL*. 2017;13:10538. https://doi.org/10.15766/mep_2374-8265.10538

Received: January 30, 2019
Accepted: June 10, 2019
Published: October 25, 2019

Copyright © 2019 Zavodnick and Kouvatsos. This is an open-access publication distributed under the terms of the Creative Commons Attribution license.
