# Supplementary material for: Electronic Health Record Skills Workshop for Medical Students
Source: MedEdPORTAL. 2019 Oct 25;15:10849. doi: 10.15766/mep_2374-8265.10849 (PMC6946580; doi:10.15766/mep_2374-8265.10849)
Supplement: Supplementary file 1 — A. Case 1.docx B. Case 2.docx C. Case 3.docx D. Student Guide.docx E. Facilitator Guide.docx F. Pretest and Posttest.docx G. EHR Presentation.pptx H. PDQI-9.pdf [file mep-15-10849-s001.zip › A. Case 1.docx]

**EHR workshop: Case 1 prompt, materials for EHR**

***Student instructions***

**Lakeisha Naylor**

You are a vascular surgeon seeing Ms. Naylor for the first time. Her chief complaint:

“I need a new surgeon now that I’ve moved to town. Claudication is still a problem for me. I’ve had lots of things done by my previous surgeon, and lots of tests too.”

**Your task:** Obtain relevant data from the EHR

Once finished, consider what information would be helpful in these alternate scenarios:

a) You are a surgeon; a patient has been referred to you for consideration of fundoplication for severe GERD

b) You are an OB-GYN; a patient establishes prenatal care with you after moving to your city at 30 weeks’ gestation

c) You are a hematologist; a patient is referred to you for anemia. They have been taking over-the-counter iron supplementation.

***EHR data***

CT angiogram aorta/femoral 4/23/2012
Exam: CTA of the abdomen, pelvis, and bilateral lower extremity runoff.
Indication: Worsening claudication
Comparison: CTA from 03/26/2012
Technique: CTA of the abdomen, pelvis, and bilateral lower extremity runoff with 3 mm axial reconstructed images following the IV administration of 115 mL of Isovue-370 IV contrast with sagittal and coronal MPR MIP images viewed on the independent workstation and saved to the PACs archive.
Findings: There has been interval development of airspace disease and atelectasis in the left lung base recommend followup imaging. The liver, spleen, pancreas, bilateral adrenal glands and kidneys have normal early arterial phase CT appearance with no focal liver lesion or hydronephrosis. The gallbladder is surgically absent. Mild degenerative changes of the lumbosacral junction. CTA findings: The abdominal aorta is normal course and caliber with mild infrarenal narrowing. The celiac axis, superior mesenteric artery, and inferior mesenteric arteries are patent. Single renal arteries bilaterally are patent. Left lower extremity runoff: The previously placed left common iliac artery stent demonstrates some peripheral mild stenosis. The origin of the internal iliac is occluded with distal reconstitution. The external iliac artery is widely patent. There is mild to moderate narrowing of the common femoral artery with extension distally. The profunda femoris artery is patent. Mild stenosis at the level of the adductor canal. The popliteal artery is widely patent. Two-vessel runoff via the posterior tibial and peroneal arteries with occlusion of the anterior tibial artery the distal calf. Right lower extremity runoff: The previously placed common iliac artery stent is widely patent. Moderate-to-severe stenosis in the proximal external iliac artery. Remainder of the external iliac artery is patent. Mild to moderate stenosis of the common femoral artery extending into the deep femoral artery. The remainder of the deep femoral artery is widely patent. Widely patent popliteal artery. High takeoff of the anterior tibial artery above-the-knee with three-vessel runoff to the ankle.
Transcribed by - DONNELL HELM
Transcription Date - XXXXXXXXXXXX

IMPRESSION:

1. Left lower extremity runoff: The previously placed left common iliac artery stent demonstrates some peripheral mild stenosis. The origin of the internal iliac is occluded with distal reconstitution. The external iliac artery is widely patent. There is mild to moderate narrowing of the common femoral artery with extension distally. The profunda femoris artery is patent. Mild stenosis at the level of the adductor canal. The popliteal artery is widely patent. Two-vessel runoff via the posterior tibial and peroneal arteries with occlusion of the anterior tibial artery the distal calf. 2. Right lower extremity runoff: The previously placed common iliac artery stent is widely patent. Moderate-to-severe stenosis in the proximal external iliac artery. Remainder of the external iliac artery is patent. Mild to moderate stenosis of the common femoral artery extending into the deep femoral artery. The remainder of the deep femoral artery is widely patent. Widely patent popliteal artery. High takeoff of the anterior tibial artery above-the-knee with three-vessel runoff to the ankle. 3. New airspace opacities and atelectasis in the left lung base and recommend followup imaging.

Note from vascular surgery, 10/24/12
PATIENT: LAKEISHA NAYLOR
HOSP NO: XXXXXXXXX
DATE OF BIRTH: 07/08/1954
ACCOUNT NO: XXXXXXXXXX
DATE OF VISIT: 10/24/2012
DICTATING PHYSICIAN: Al Beaty, M.D.
STAFF PHYSICIAN: Quinton Schroeder, M.D.
VASCULAR SURGERY CLINIC - REEVALUATION

HISTORY OF PRESENT ILLNESS: This is a 58-year-old woman who has a history of coronary artery disease, hypertension, hyperlipidemia,fibromyalgia, chronic obstructive pulmonary disease (COPD), and peripheral vascular disease as well as a left knee meniscal tear. She has had difficulty with lower extremity pain and weakness with walking and had a lower extremity arterial Doppler on 02/29/2012 that showed a right ankle-brachial index (ABI) of 0.55 and a left ABI of 0.9. On 06/19/2012, she underwent right external iliac stenting which improved her left lower extremity ABI to 0.72; however, she did not experience improvement in her symptoms. She returns to clinic now after having had repeat lower extremity arterial Doppler and for further consultation.

Her right lower extremity ABI is now 0.57, and her left is 0.84, which is close to her preoperative baseline. She still complains of severe aching pain in both of her thighs and legs when she stands for a long period of time or walks a short distance. She also feels like her legs get very weak after walking; therefore, she drives around in a motorized scooter for the most part. She has indeed quit smoking ever since her revascularization surgery in 06/22/2012; however, she still has frequent and powerful cravings for cigarettes.

PHYSICAL EXAMINATION:
Vital Signs: Heart rate 91, blood pressure 120/66.
General: Awake, alert, in no acute distress. Morbidly obese woman lying on the examining table.
HEENT: Normocephalic, atraumatic.
Neck: Supple.
Heart: Regular rate and rhythm.
Lungs: She is visibly short of breath, which she says is her baseline from COPD. No audible wheezing.
Abdomen: Obese, nontender.
Extremities: She has dorsalis pedis and posterior tibial pulses bilaterally. No open wounds. She has 1+ pitting edema in her legs bilaterally.

DIAGNOSTIC DATA: Lower extremity arterial Doppler from 10/24/2012, preliminary report, right ankle-brachial index is 0.57, left ankle-brachial index is 0.84.

ASSESSMENT AND PLAN: This is a 58-year-old woman with multiple medical comorbidities with peripheral artery disease and moderately reduced arterial supply to her right lower extremity.

1. Because she experienced no improvement in her symptoms after revascularization and because her symptoms are bilateral and equal despite the discrepancy in ABI between the right and left leg, it is very unlikely that the limiting factor in her mobility and lower extremity pain is vascular in nature. The history she gives including severe aching back pain with similar aching pain in her thighs bilaterally associated with weakness
with standing and walking is more consistent with pseudoclaudication or neurogenic claudication. She has been told in the past that she has degenerative disc disease but has not seen a neurologist or a neurosurgeon in the recent past. We have made a referral to General Surgery as well as Neurology for her chronic back pain and lower extremity weakness and pain.
2. For the smoking cessation, the patient quit smoking four months ago but still has strong cravings. We have written her a prescription for Chantix in hopes that this will reduce her cravings and reduce the rate of relapse into smoking. Because of her extensive polypharmacy, a drug interaction review was undertaken, and no drug interactions with Chantix were found. She is to call the clinic in a month to obtain a
refill of her Chantix for maintenance therapy.
3. Return to clinic in three months for re-evaluation.

STAFF PHYSICIAN REVIEW: The patient was examined and discussed with
Quinton Schroeder, M.D.

ATTENDING ADDENDUM:
I evaluated the patient and reviewed the history and physical exam as documented by the resident. We also reviewed the pertinent studies and imaging with the following notable findings:
1.) s/p left EIA stent with brief improvement in ABI, now back down to 0.57
2.) patient non-ambulatory likely secondary to neurogenic claudication
I discussed with the patient their vascular diagnosis as listed above, including our expected management and follow-up plan of which they were agreeable. This involves neurology evaluation for neurogenic etiology of back and LE pain.

PCP note 3/20/2013
58 y/o female here for f/u DM2

HPI
only taking some of medications every other day because of new increased spend down amount because of income changes. leg pain continues, had mri which is negative. missed appt with vascular surgery because upset that they can't fix leg pain that has been ongoing for years. no changes in right leg pain, but is more severe since has been trying to conserve pain medication. pain is an ache and sharp down entire right leg. activity increases pain. continues to smoke 1/2 ppd. does not check glucose at home, despite having supplies.

PMH
HTN
COPD
fibromyalgia
chronic back pain
PVD s/p right proximal ext arterial iliac stent 06/22/2012-ABI 10/22/2012 R-0.57, L-0.84 no change, stents patent
colon 2008
mammo 2011

Meds
lisinopril 20 mg po qd
nexium 20 mg po qd
paxil 60 mg po qd
flonase
gemfibrizol 600 mg po bid
glipizide xl 20 mg po qd
ibuprofen prn
hydrocodone 5/500mg #240
alprazolam 0.25mg tid #84
baclofen 10 mg po tid
plavix 75 mg po qd
premarin
amlodipine 5 mg po
lyrica 150 mg po bid
asa 325 mg po qd
promethazine 25 mg po prn
advair
olopatadine 0.1
aviva strips
nystatin powder and suspension
albuterol
cilostazol

ROS
+weight loss, fatigue, chronic leg pain, depression, anxiety
all other systems reviewed and negative

O:
Vitals: 127/73
General: NAD, alert and pleasant
HEENT: EOMI, TM clear, no dc ext canals, no erythema and OP clear
Neck: Neck supple, Thyroid midline and No masses
CV: RRR and No murmur
Pulm: CTAB, no wheeze, rales and crackles
Abd: soft, round, nontender, nondistended and + bowel sounds
Ext: MAE, no edema, no cyanosis and no clubbing
Foot exam: bilateral- no ulcers, lesions, deformities. 1+dp pulses, nails good repair.

A and P:

diabetes mel non insulin dep, not at goal. A1c today 8.5% increased from 8%
counseled pt on diet changes, if ineffective will start insulin at next visit
encouraged checking glucose and medication adherence to prevent negative health effects

HBP essential, at goal
continue current medical regimen

leg pain, right chronic. mri negative for lumbar djd, abnormalities
continue current pain medications
encouraged pt to f/u with vascular surgery for further eval of pain as missed appt last month

social prob othr, acute
referred pt to Asia Wagoner social worker

ABI/dopplers 1/29/14
Ms. LAKEISHA NAYLOR had a Lower Limb Arterial Duplex, Complete Bilateral on 01/29/2014. If you have any questions regarding this study please do not hesitate to contact us.
Indications: Bilateral Atherosclerosis with Claudication [440.21].
Risk Factors: The patient admits to a positive history of hypertension.
Clinical Examination: Bilat claud. Hx of EIA stents.

Conclusion:
Right leg presents with mildly diminished distal perfusion at rest. ABI = 0.63 with biphasic flow at ankle.
Left leg presents with normal distal perfusion at rest. ABI = 1+ with triphasic flow at ankle.
Bilateral external iliac arteries have strong pulses through the stents.
Prior test 10/24/2012 = Right ABI = 0.57 and Left ABI = 0.84.
