# Supplementary material for: Electronic Health Record Skills Workshop for Medical Students
Source: MedEdPORTAL. 2019 Oct 25;15:10849. doi: 10.15766/mep_2374-8265.10849 (PMC6946580; doi:10.15766/mep_2374-8265.10849)
Supplement: Supplementary file 1 — A. Case 1.docx B. Case 2.docx C. Case 3.docx D. Student Guide.docx E. Facilitator Guide.docx F. Pretest and Posttest.docx G. EHR Presentation.pptx H. PDQI-9.pdf [file mep-15-10849-s001.zip › B. Case 2.docx]

**EHR workshop: Case 2 prompt, materials for EHR**

***Student instructions***

**Roger Conwell**

You are the internal medicine intern taking over Mr. Conwell’s hospital care. He was admitted on 7/7/2012; there was EHR downtime on 7/8, and the paper progress note was lost. Today is 7/9/2012. You receive handoff from the departing intern:

“Mr. Conwell, he’s stable. He was admitted for hypotension from diarrhea. Things came back negative, it was probably a gastroenteritis. He had a lot of diarrhea but that’s resolved now, and his blood pressure improved with fluid. He should probably go home soon. There were some other little issues, my admission note is pretty detailed.”

**Your tasks:**

1) Write a progress note for 7/9/2012 (use Word or similar – no note-writing function in tEMR). *You may BEGIN by copy/paste, but make sure every word is accurate/updated, and that sentences reflecting thought processes are your own (vs. objective findings which are OK as long as they are accurate).*
2) Create a list of tasks that need to be accomplished today

***EHR data***

Admission note 7/7/12

ICU Admission History and Physical

Chief Complaint: Confusion

History of Present Illness:
Mr. Conwell is a 59yo WM with pmh significant for DVT/PE (on chronic anticoagulation), DM, HTN, h/o diverticulitis who presents from home with complaints of confusion. Patient has been feeling extremely fatigued and malaise for at least last 2 weeks. Sleeping all the time. He saw PCP recently and she gave him an antibiotic which he took one of yesterday. Yesterday, he reports 10 bouts of profuse watery diarrhea. This morning another 2-3 episodes. No black/bloody/tarry stools. He was later cleaning in the kitchen when he felt very light headed and started seeing spots. His son brought him to the ED. He also reports not being on his Arixtra for last 1 week.
In the ED, patient found to have SBP approx 70's, POX 88% RA, Pulse 40's. He was given fluids. Because of concern for DVT dopplers were performed and did not demonstrate DVT. Cardiology was called for stat echo, concern for R heart strain, but this was not demonstrated. He was continued on aggressive intravenous hydration and transferred to ICU for further management.

Past Medical History (per EMR and patient):
1. DVT/PE
2. Diabetes
3. Hypertension
4. Dyslipidemia
5. Chronic Pain
6. Diverticulitis
7. Renal Calculi

Past Surgical History:
numerous including -
- open cholecystectomy
- appendectomy
- abdominal hernia
- hip and knee surgeries

Medications (per patient):
1. Insulin Novolog 10 units + SSI TID
2. Insulin Lantus 40 units qHS
3. Simvastatin 40mg qHS
4. Lisinopril 40mg daily
5. Metoprolol 25mg BID (had not taken day of admit)
6. Arixtra 10mg daily (had not taken for 1 week prior to admit)
7. MSSR 30mg BID
8. Percocet q4-6hours PRN
9. Gabapentin 600mg TID
10. Nexium 40mg daily
11. Flonase
12. Aspirin 81mg

Allergies:
- Vancomycin - swelling
- Clindamycin - swelling
- Ceftriaxone - swelling
- Fenoprofen
- Betadine/Topical Iodine

Social History: No illicit drug use, tobacco, or alcohol use. Wife recently passed away earlier this year from cancer.

Family History: father with lymphoma

Physical Exam:
Vitals - afebrile, P55, BP 80/60, R16, POX 95% 2L
General - obese WM lying in bed, NAD, A+Ox3, drowsy
HENT - NC/AT, dry MM, OP clear
Eyes - PERRLA, EOMI, no scleral icterus or conjunctival injection
Neck - no cervical LAD, normal thyroid, unable to appreciate JVD
Heart - borderline bradycardic, regular rhythm, no R/G/M
Lungs - scattered wheezes, good aeration otherwise
Abdomen - obese, some pain to palpation over R hernia site (old pain per patient)
Ext - no LE edema, no redness/erythema, Homan's sign negative
Skin - some bruising over R thigh (where he injects insulin), open chole scar, R knee surgical scar
Neuro - CN II-XII grossly intact, no focal neuro deficits
Psych - appropriate mood and affect

Labs - reviewed

Imaging - reviewed

EKG - sinus bradycardia. no heart block. no heart strain.

A/P:
59yo WM with pmh significant for DVT/PE (on chronic anticoagulation), DM, HTN, h/o diverticulitis who presents from home with complaints of confusion. Found to be hypertensive, bradycardic, with worsening of renal function.

1. Hypotension and diarrhea - likely hypotensive from hypovolemia 2/2 diarrhea. Will watch H/H, no evidence of bleeding but high risk due to anticoagulation. Blood pressure has responded to 6L IVF. Lower suspicion for bacterial infection - no fever, elevated WBC. Would be worth investigating etiology of diarrhea
- C. Diff, stool culture
- RSV, influenza screen
- Broad spectrum antibiotics while awaiting blood cultures, urine culture - if these are negative will discontinue
- Hold all antihypertensives for now
- Continue MIVF
- Awaiting echo report, by verbal report no RH strain
- Will make sure to repeat CBC today

2. Bradycardia - unclear cause. EKG demonstrates only sinus rhythm, no heart strain. Initial set of cardiac markers negative. Did have episode of chest discomfort earlier today that resolved. Reportedly did not take metoprolol today. Possibly from MSSR? Electrolytes normal. Nevertheless, bradycardia has resolved with fluids. Cardiac echo normal.
- Monitor on telemetry
- Serial cardiac markers
- If troponin positive, or if CK >200, will page cardiology STAT

3. Confusion - likely from hypotension as above. ETOH negative. Ammonia normal. Patient back to baseline per son.
- Neurochecks

4. DVT/PE - has been off arixtra for at least 1 week. No DVT per LE doppler. No s/s of PE.
- Will likely restart in next day if stable, no evidence of bleeding

5. Cystitis
- Today is day 4/14 of Cipro prescribed by PCP

6. Diabetes - poorly controlled per A1c earlier this year. Some episodes of hypoglycemia so far. Not taking much PO currently.
- Recheck HgA1c
- Hold lantus
- SSI

7. h/o Hypertension - hold antihypertensive given hypotension as above

8. Dyslipidemia - continue home statin

9. Chronic Pain - no current complaints.
- Continue gabapentin
- Hold off on narcotics

10. FEN/GI - diabetic diet. Home PPI.

11. Ppx - SCDs

12. Patient is FULL CODE

13. Dispo - admit to MICU for close observation. Can likely transfer to PICU tomorrow if stable.

Lab results for 7/7/12
CBC: White blood count 4.8 k/cumm, hemoglobin 12.9 g/dL, platelets 198 k/cumm
Troponin <0.05ng/mL at 02:00, 11:00
CK 261 units/L at 0:200, 194 units at 11:00
Hemoglobin A1C 8.4%

Echocardiogram for 7/7/12
CONCLUSION:
Left ventricular cavity size normal.
Normal left ventricular systolic function.
Abnormal relaxation filling pattern of the left ventricle (Stage 1 diastolic dysfunction).
No obvious regional wall motion abnormalities.
Mild left atrial dilatation.
Normal right atrial size.
Normal right ventricular global systolic function.
Right ventricular systolic pressure cannot be accurately estimated.
No significant valve abnormalities. Pulmonic valve not well visualized.
No pericardial effusion.

Vitals for 7/9/12
Temperature 98.7, pulse 85, blood pressure 166/94, respirations 12, oxygen saturation 93% on room air

Lab results for 7/9/12
8am blood glucose: 335
Stool culture and clostridium difficile: negative
