# Supplementary material for: Electronic Health Record Skills Workshop for Medical Students
Source: MedEdPORTAL. 2019 Oct 25;15:10849. doi: 10.15766/mep_2374-8265.10849 (PMC6946580; doi:10.15766/mep_2374-8265.10849)
Supplement: Supplementary file 1 — A. Case 1.docx B. Case 2.docx C. Case 3.docx D. Student Guide.docx E. Facilitator Guide.docx F. Pretest and Posttest.docx G. EHR Presentation.pptx H. PDQI-9.pdf [file mep-15-10849-s001.zip › C. Case 3.docx]

**EHR workshop: Case 3 prompt, materials for EHR**

***Student instructions***

**Lucindo Zarate**

You are a pediatrician. Your partner has gone on vacation and you are receiving all lab results for their patients. Today is 10/17/2018, and you have received an EHR alert for Lucinda’s comprehensive metabolic panel with some abnormal values.

**Your tasks:**

1) Gather information to explain these findings
2) Communicate results to patient’s parent, and document your conversation (in Word or similar; no note-writing functionality in tEMR)
3) Based on your conversation with the parent, develop a management plan, placing orders if appropriate

***EHR data***

Progress note from 10/2
Presenting problem: Mom, Sheena Hackett, states, “He’s had diarrhea for the past two weeks now – I’ve changed his diet to bland foods and solid foods – he pretty much doesn’t eat anymore – I’ve been giving him Pedialyte. They said to call this week if he still had diarrhea to get in for an appointment.”

Call center assessment: CC: diarrhea Onset/Duration: started 12/17 and has been daily - mostly at night Location: GI Severity/Description: had diarrhea 4 small to large watery stools yesterday - sts. stools are watery brown with gravelly sand-type material Pain Assessment: denies acting like he is having pain anywhere except for diaper rash on bottom Play Activity: active, playful Hydration status: taking fluids well & urinating at least three times per day; mouth moist inside assoc. Symptoms: has vomited 3x in past 2 wks - last time was 12/29; taking liquids well, but not eating solid foods much - took small amount of oatmeal this morning; has large diaper rash on both buttocks with some blistering, but blisters are intact - has spread down to legs a little; has been more tired lately - admits he is up during the night for diaper changes and not sleeping as well due to this Short Term Meds.: aveeno diaper rash ointment; baby powder PMHx: denies Allergies: NKDA Sts. they do have a snake, but have had it for several years and it is not new.

Labs from 10/16
Comprehensive metabolic panel: sodium 139 mmol/L, potassium 2.7 mmol/L, chloride 107 mmol/L, CO2 19 mmol/L, anion gap 13 mmol/L, BUN 7 mg/dL, creatinine 0.5 mg/dL, glucose 94 mg/dL, calcium 9.2 mg/dL, albumin 4.3 mg/dL, bilirubin 0.3 mg/dL, Alk Phos 234 mg/dL, AST 31 IU/L, ALT 21 IU/L, protein 7.1 g/dL
