# Supplementary material for: Electronic Health Record Skills Workshop for Medical Students
Source: MedEdPORTAL. 2019 Oct 25;15:10849. doi: 10.15766/mep_2374-8265.10849 (PMC6946580; doi:10.15766/mep_2374-8265.10849)
Supplement: Supplementary file 1 — A. Case 1.docx B. Case 2.docx C. Case 3.docx D. Student Guide.docx E. Facilitator Guide.docx F. Pretest and Posttest.docx G. EHR Presentation.pptx H. PDQI-9.pdf [file mep-15-10849-s001.zip › D. Student Guide.docx]

Using the electronic health record workshop

XXXXX X, 20XX

**Case 1**

**Lakeisha Naylor**

You are a vascular surgeon seeing Ms. Naylor for the first time. Her chief complaint:

“I need a new surgeon now that I’ve moved to town. Claudication is still a problem for me. I’ve had lots of things done by my previous surgeon, and lots of tests too.”

**Your task:** Obtain relevant data from the EHR

Once finished, consider what information would be helpful in these alternate scenarios:

a) You are a surgeon; a patient has been referred to you for consideration of fundoplication for severe GERD

b) You are an OB-GYN; a patient establishes prenatal care with you after moving to your city at 30 weeks’ gestation

c) You are a hematologist; a patient is referred to you for anemia. They have been taking over-the-counter iron supplementation.

**Case 2**

**Roger Conwell**

You are the internal medicine intern taking over Mr. Conwell’s hospital care. He was admitted on 7/7/2012; there was EHR downtime on 7/8, and the paper progress note was lost. Today is 7/9/2012. You receive handoff from the departing intern:

“Mr. Conwell, he’s stable. He was admitted for hypotension from diarrhea. Things came back negative, it was probably a gastroenteritis. He had a lot of diarrhea but that’s resolved now, and his blood pressure improved with fluid. He should probably go home soon. There were some other little issues, my admission note is pretty detailed.”

**Your tasks:**

1) Write a progress note for 7/9/2012 (use Word or similar – no note-writing function in tEMR). *You may BEGIN by copy/paste, but make sure every word is accurate/updated, and that sentences reflecting thought processes are your own (vs. objective findings which are OK as long as they are accurate).*
2) Create a list of tasks that need to be accomplished today

**Case 3**

**Lucindo Zarate**

You are a pediatrician. Your partner has gone on vacation and you are receiving all lab results for their patients. Today is 10/17/2018, and you have received an EHR alert for Lucinda’s comprehensive metabolic panel with some abnormal values.

**Your tasks:**

1) Gather information to explain these findings
2) Communicate results to patient’s parent, and document your conversation (in Word or similar; no note-writing functionality in tEMR)
3) Based on your conversation with the parent, develop a management plan, placing orders if appropriate
