# Supplementary material for: Electronic Health Record Skills Workshop for Medical Students
Source: MedEdPORTAL. 2019 Oct 25;15:10849. doi: 10.15766/mep_2374-8265.10849 (PMC6946580; doi:10.15766/mep_2374-8265.10849)
Supplement: Supplementary file 1 — A. Case 1.docx B. Case 2.docx C. Case 3.docx D. Student Guide.docx E. Facilitator Guide.docx F. Pretest and Posttest.docx G. EHR Presentation.pptx H. PDQI-9.pdf [file mep-15-10849-s001.zip › E. Facilitator Guide.docx]

EHR workshop facilitator guide

Facilitator should have access to this guide as well as a copy of the student instructions for each case. For timekeeping, you may find it useful to list actual times rather than durations in the “time” column.

| Time | Activity | Facilitator activities |
| --- | --- | --- |
| 15 minutes | Sign in  Pretest  Introduction/slides | Attendance as appropriate for your course as students enter  Administer pretest - since questions are identical to posttest, we passed out a pretest/posttest packet, each with a matching number (e.g. student 1 gets a pretest and posttest, both labeled 1) which enabled assessing an individual student’s pre-post performance. Could collect pretest right away, or use honor system. Allow about 5 minutes, less if students appear to have finished.  Deliver slides introducing knowledge, concepts, and structure of the activity – talking points are in the notes section |
| 20 minutes | Small group work on cases | Form groups of about 4 students each  Assign one case to each group (e.g. one group works on case 1, the next group works on case 2, etc)  Students should bring an internet-capable laptop or computer. If you administer the workshop using a training environment in your EHR, devices should be able to access this.  Case 2 will require word processing abilities in addition to EHR access |
|  | Case 1 | Students are asked to “obtain relevant data from the EHR”. In this situation, this includes the note from vascular surgery 10/24/12; scanty vascular surgery note but also ABI/dopplers 1/29/14. It might be reasonable to include aorta/fem CT angiogram from 4/23/2012 (but this imaging is prior to most recent intervention, so it would also be reasonable to consider it irrelevant).  A useful discussion question as you observe the groups is “how did you approach the problem?” Steer groups toward a focused search rather than gathering “everything” – if their list of “relevant data” includes every note from the past few years, point out that there is a *lot* of data there, much of it pertaining to medical problems not being actively addressed, and it might take longer to wade through all that than to take a few minutes to decide what information to seek. “Get all the records” is not a solution – there are just too many! If students haven’t yet experienced obtaining outside records, inform them that they will be asked to fill out a form stating specifically which records they want in a way understandable to medical records personnel.  If groups have time to do the alternate cases, here is the data that they should find relevant:  a) Surgeon: prior GI or PCP notes describing GERD, prior upper endoscopy, esophageal pH monitoring, manometry, prescription history  b) OB-GYN: office notes from last prenatal, other pregnancies, delivery note from prior pregnancies, prenatal labs incl CBC, HIV; ultrasound reports  c) Hematologist: prior iron studies, reticulocytes, B12/folate, hemoglobin electrophoresis; colonoscopy; GYN notes or pelvic US if female; PCP note or hospital DC summary if any reason to suspect anemia of chronic inflammation  Other possible discussion points: these should be used to guide the large-group discussion when the groups present their findings, so that all students get exposed to discussion points for all cases. However, they may be helpful in directing groups that are struggling with the exercise, so they are included here.  Prompt them to describe why they start their search where they did. Dig deeper – “vascular surgery note because it’s a vascular problem” just scratches the surface, where “vascular surgery note because it might succinctly list prior procedures and refer to or even include specifics on recent imaging” is a more thoughtful approach. A PCP’s note might frame the problem overall or summarize prior treatment for the complaint. This might be useful (as a cardiologist, a patient being referred for chest pain might have a PCP note describing their multiple coronary risk factors; or describing a likely diagnosis of costochondritis but the patient’s extreme worry about coronary disease which prompted the consultation) – but try to balance building on prior work with avoiding early diagnostic closure.  Discuss how EHR “habits” might work for special situations (e.g. a vascular surgeon could always look for the same things); generalists and new problems merit a more tailored approach problem by problem.  Discuss the concept of EHR “rumors” – inaccurate information that, once documented in the EHR, can be passed along (or even copy-pasted) unquestioned, leading to the persistence of incorrect diagnoses. I shared the story of my grandfather who somehow obtained a label of “Parkinson’s Disease” in the EHR despite having no symptoms and no doctor who had made this diagnosis, many who agreed it was unlikely – and tried unsuccessfully for years to have it removed from his record. Ask students to reflect how likely they are to question a diagnosis that doesn’t seem to fit, and whether that diagnosis appearing in multiple EHR notes would affect this. In this acse, the primary care (PCP) note from 3/20/2013 says “missed appt with vascular…because upset that they can’t fix leg pain” – consider this against reality of vascular surgery note 10/2012 which suggests that the pain was unaffected by revascularization, and the surgeon arranged for surgery and neurology referrals to treat degenerative disk disease as a more likely cause of her symptoms? Do you think the PCP’s understanding reflects the patient’s perception of the vascular appointment? How will patient note access affect the accuracy of these notes (encourage them to think of examples where accuracy may improve, e.g. patients correcting errors/”rumors,” and where it may suffer, e.g. doctors hesitating to give their thoughts on somatization, the role of lifestyle factors, etc)? |
|  | Case 2 | Students are given an admission note from 7/7/12 and asked to write a progress note from 7/8/12.  Hypotension presumed from diarrhea was main problem; note mentions need to check C diff, empiric broad spectrum antibiotics until negative blood cultures, holding antihypertensives, and checking echo. Students are then given a c diff result, an elevated blood pressure, and an echo result which should result in appropriate changes to this item on the plan.  Bradycardia was problem #2; on H&P, plan made to page cardiology if elevated troponin or CK; students are given these results as normal and this should be made clear in the progress note they write.  Problem #4: history of DVT/PE, anticoagulation being held but with plans to restart if no evidence of bleeding. Students are given a stable hemoglobin result and should likely update this plan to restart.  Problem #5: cystitis, on day 4/14 of antibiotics at time of admission. That would make the day of the progress note day #6 of antibiotics. It would be appropriate to question a 14-day course of antibiotics for what appears to be uncomplicated cystitis, but the group should not derail the discussion too far into this area.  Problem #6: diabetes, long acting insulin being held on admission, receiving sliding scale only. Students are given AM blood glucose of 335 and should revisit this plan.  Problem #6: hypertension, holding antihypertensives. This is mentioned in problem #1 as well; careful groups will update their plan in both areas to resume at least one antihypertensive. Ask groups if they’ve seen internally inconsistent notes like the one that would result if only problem #1 was updated.  **It will be useful in the large-group discussion to have a projected or handout version of the notes they create. Facilitators should ask them to e-mail the note to facilitators, or print a copy, or use a flash drive, whatever makes sense with the tools available. This can also be added to the student instructions.**  Other possible discussion points: these should be used to guide the large-group discussion when the groups present their findings, so that all students get exposed to discussion points for all cases. However, they may be helpful in directing groups that are struggling with the exercise, so they are included here.  Are there any if-thens in the note that helped inform your plan for the day? (If no evidence of bleeding resume anticoagulation; if blood and urine cultures negative, stop antibiotics; if troponin positive, page cardiology)  What would you tell the PCP about this hospital course, if you were to call them upon discharge today? This prompt tests their ability to prioritize  What do you think about the use of “antibiotic day 4/14?” Have you seen this done another way? A preferred method now that copy-paste is common is to write “a 14-day course of cipro, last day 7/17” which is accurate even if copied forward. |
|  | Case 3 | Students are contacted with abnormal lab results (hypokalemia, metabolic acidosis) from a patient they have never seen. They are asked to gather enough information to explain the results, to communicate these results to the patient’s parent and in an EHR note, and to develop a plan for management of the abnormal findings.  An EHR search will reveal info: **two** progress notes from 10/2; one is a phone call from the patient’s mother, Sheena Hackett, which includes the context explaining why labs were drawn; labs from 10/16 are also available.  Once students understand why labs were ordered, they should understand the importance of reaching out to the patient’s mother. When they do so, the facilitator can respond with one of the following scripts (can vary between groups, or if only one group ask them to respond separately to each scenario).  **Parent script A**: “Lucindo is still vomiting and having diarrhea. He is very lethargic, has a high fever, and hasn’t eaten in days. His lips are dry and cracked.”  Expected response from students: go to ED. This should be communicated to patient’s mother and documented in an EHR note (or word processing program if EHR lacks note writing capabilities)  **Parent script B:** “Lucindo stopped vomiting the day after we had those labs drawn! He’s himself now, happy and playful – he’s eating better though still not totally normal yet. [If asked] We definitely don’t want to come into the office or the hospital, we live very far away.”  Expected response from students: order repeat labs, potassium supplement, and communicate this to mother as well as writing note in EHR (or word processing program)  If students at your institution don’t routinely place orders, this is a good opportunity for them to try ordering a BMP and potassium supplements. They should feel free to use any resources available to look up appropriate dosing.  Other possible discussion points: these should be used to guide the large-group discussion when the groups present their findings, so that all students get exposed to discussion points for all cases. However, they may be helpful in directing groups that are struggling with the exercise, so they are included here.  What is your responsibility when unsolicited information is routed to you? Does this depend on the acuity of the information? Does it depend on how comfortable you are with the indication for the test (e.g. TSH for thyroid replacement monitoring if you are very familiar with this, versus if you rarely order this test)? What about the seriousness of the suspected findings (e.g. a chest X-ray with a nodule in a patient with a long smoking history)? How does it change things to have access to a list of other recipients?  If you are the only recipient of a critical result for a patient you’ve never met, under what circumstances should you reach out to the patient? Likely very few – patients deserve results from a doctor familiar with why they were ordered and what they mean, and the doctor with appropriate context will be better able to dire, so reaching out to a clinician is usually more appropriate. That said, there may be emergency situations where this is not possible, or situations where you are “covering” for the more appropriate clinician and you need to make a decision to try to reach them or reach the patient directly. |
| 15 minutes | Discuss case 1 | Ask team(s) to briefly describe task and how they accomplished it; cover the discussion points listed under the case above. |
| 15 minutes | Discuss case 2 | **If possible, have the groups’ notes projected (email to faculty, provide flash drives) or print multiple copies to pass out.** You may want to add these instructions to the student guide for case 2.  If this is possible, use the PDQI-9 (see slide deck) to assess the quality of the note.  If projection and handouts are not feasible, direct groups who were assigned case 2 to assess their notes using the PDQI-9 (project this slide or provide handouts), while groups not assigned case 2 should be directed to the H&P and use the PDQI-9 to assess that. Provide just a minute or two for small groups to work on this; a completed PDQI-9 “score” from each group is less important than a discussion of the elements of the score, what features of the notes are better or worse as defined by this tool, and whether that feels clinically appropriate.  If time allows, cover the discussion points listed under the case above. |
| 15 minutes | Discuss case 3 | Ask team(s) to briefly describe task and how they accomplished it; cover the discussion points listed under the case above. |
| 10 minutes | Wrap-up | Administer post-test and discuss results  What are the main take-home points (elicit from group)? |
