# Supplementary material for: Electronic Health Record Skills Workshop for Medical Students
Source: MedEdPORTAL. 2019 Oct 25;15:10849. doi: 10.15766/mep_2374-8265.10849 (PMC6946580; doi:10.15766/mep_2374-8265.10849)
Supplement: Supplementary file 1 — A. Case 1.docx B. Case 2.docx C. Case 3.docx D. Student Guide.docx E. Facilitator Guide.docx F. Pretest and Posttest.docx G. EHR Presentation.pptx H. PDQI-9.pdf [file mep-15-10849-s001.zip › F. Pretest and Posttest.docx]

Using the electronic health record pretest

1. The purpose of the medical note has always included to describe symptoms and define diseases.
   1. True
   2. **False**
2. With the evolution of the medical note, the purpose now also includes:
   1. To describe how sick patients are to start with
   2. To bill for level of service
   3. To educate patients
   4. a and b
   5. **All of the above**
3. When writing a note, you can copy & paste portions of another author’s note if true to the patient’s condition and your assessment.
   1. True
   2. **False**
4. When gathering information regarding a particular medical problem for a patient, the last PCP note should always be the first note to review.
   1. True
   2. **False**
5. You receive an abnormal lab result in your inbox for a patient who is not under your care. You should:
   1. Review the result and mark it complete
   2. Send it to the ordering physician
   3. **Send it to the ordering physician and request confirmation of receipt**
   4. Call the patient and discuss the abnormal finding
6. I feel confident querying the EHR in an efficient, complete manner.
   1. Strongly Agree
   2. Agree
   3. Neutral
   4. Disagree
   5. Strongly Disagree
7. I feel confident creating documentation, avoiding common mistakes, in the EHR.
   1. Strongly Agree
   2. Agree
   3. Neutral
   4. Disagree
   5. Strongly Disagree
8. I feel confident initiating management plans from results obtained from the EHR.
   1. Strongly Agree
   2. Agree
   3. Neutral
   4. Disagree
   5. Strongly Disagree

Using the electronic health record posttest

1. The purpose of the medical note has always included to describe symptoms and define diseases.
   1. True
   2. **False**
2. With the evolution of the medical note, the purpose now also includes:
   1. To describe how sick patients are to start with
   2. To bill for level of service
   3. To educate patients
   4. a and b
   5. **All of the above**
3. When writing a note, you can copy & paste portions of another author’s note if true to the patient’s condition and your assessment.
   1. True
   2. **False**
4. When gathering information regarding a particular medical problem for a patient, the last PCP note should always be the first note to review.
   1. True
   2. **False**
5. You receive an abnormal lab result in your inbox for a patient that is not under your care. You should:
   1. Review the result and mark it complete
   2. Send it to the ordering physician
   3. **Send it to the ordering physician and request confirmation of receipt**
   4. Call the patient and discuss the abnormal finding
6. I feel confident querying the EHR in an efficient, complete manner.
   1. Strongly Agree
   2. Agree
   3. Neutral
   4. Disagree
   5. Strongly Disagree
7. I feel confident creating documentation, avoiding common mistakes, in the EHR.
   1. Strongly Agree
   2. Agree
   3. Neutral
   4. Disagree
   5. Strongly Disagree
8. I feel confident initiating management plans from results obtained from the EHR.
   1. Strongly Agree
   2. Agree
   3. Neutral
   4. Disagree
   5. Strongly Disagree

Would you recommend this workshop be offered in future Gateway courses?

If no, would you be more likely to recommend this course if our local EHR was used?

Feedback/Suggestions:
