# Supplementary material for: Electronic Health Record Skills Workshop for Medical Students
Source: MedEdPORTAL. 2019 Oct 25;15:10849. doi: 10.15766/mep_2374-8265.10849 (PMC6946580; doi:10.15766/mep_2374-8265.10849)
Supplement: Supplementary file 1 — A. Case 1.docx B. Case 2.docx C. Case 3.docx D. Student Guide.docx E. Facilitator Guide.docx F. Pretest and Posttest.docx G. EHR Presentation.pptx H. PDQI-9.pdf [file mep-15-10849-s001.zip › G. EHR Presentation.pptx]

## Slide 1
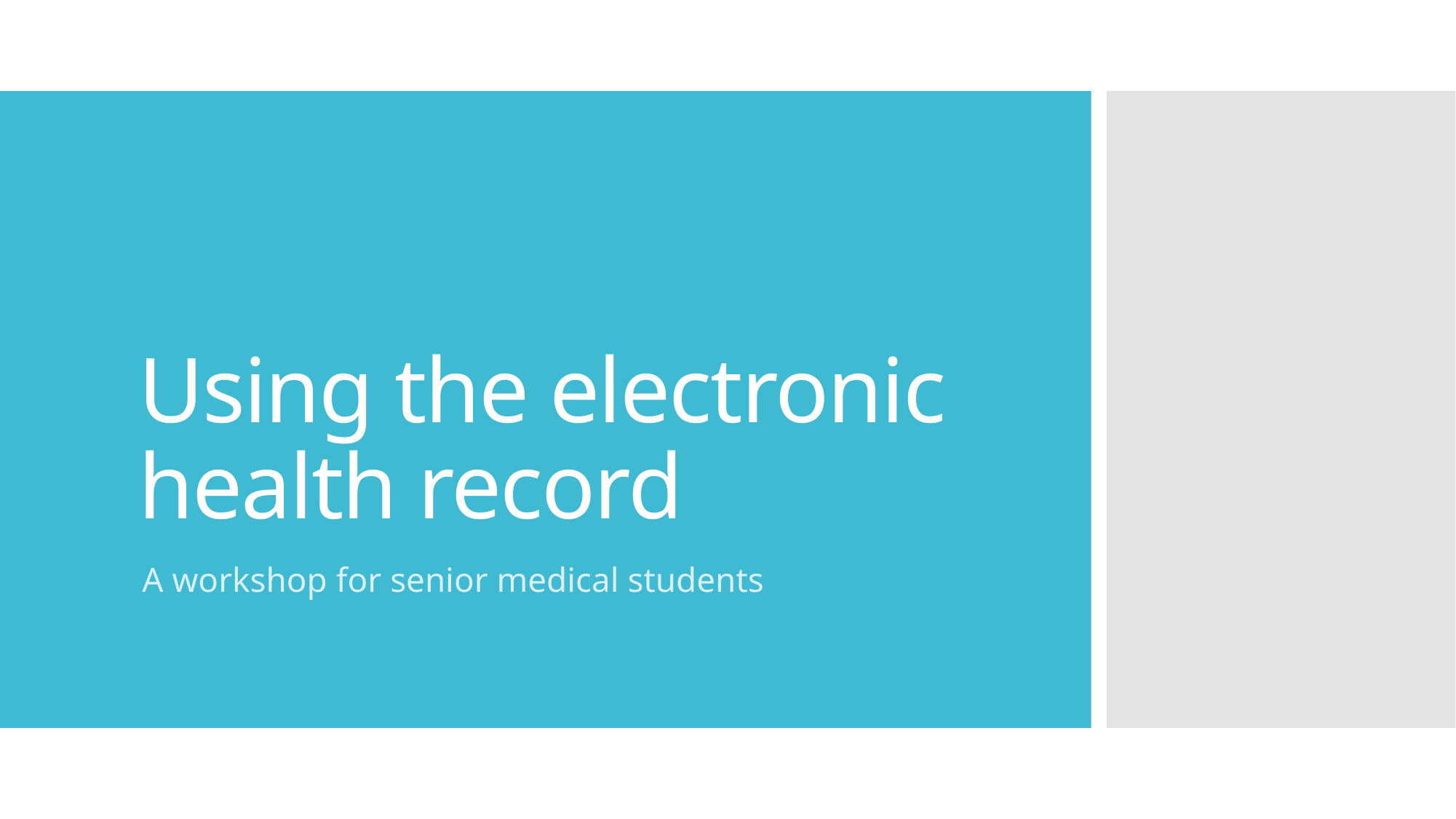

# Using the electronic health record
A workshop for senior medical students

## Slide 2
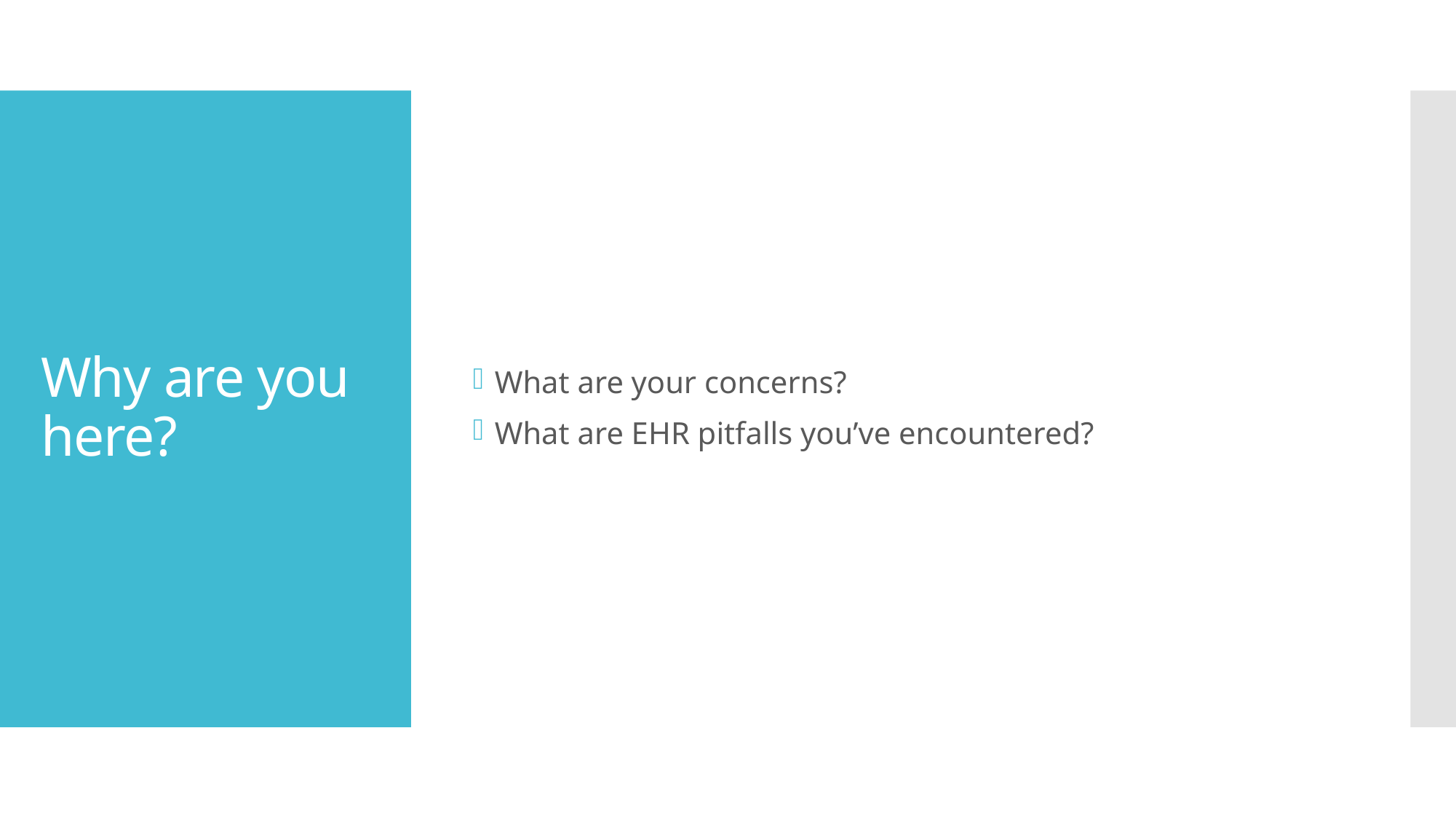

What are your concerns?
What are EHR pitfalls you’ve encountered?
# Why are you here?

## Slide 3
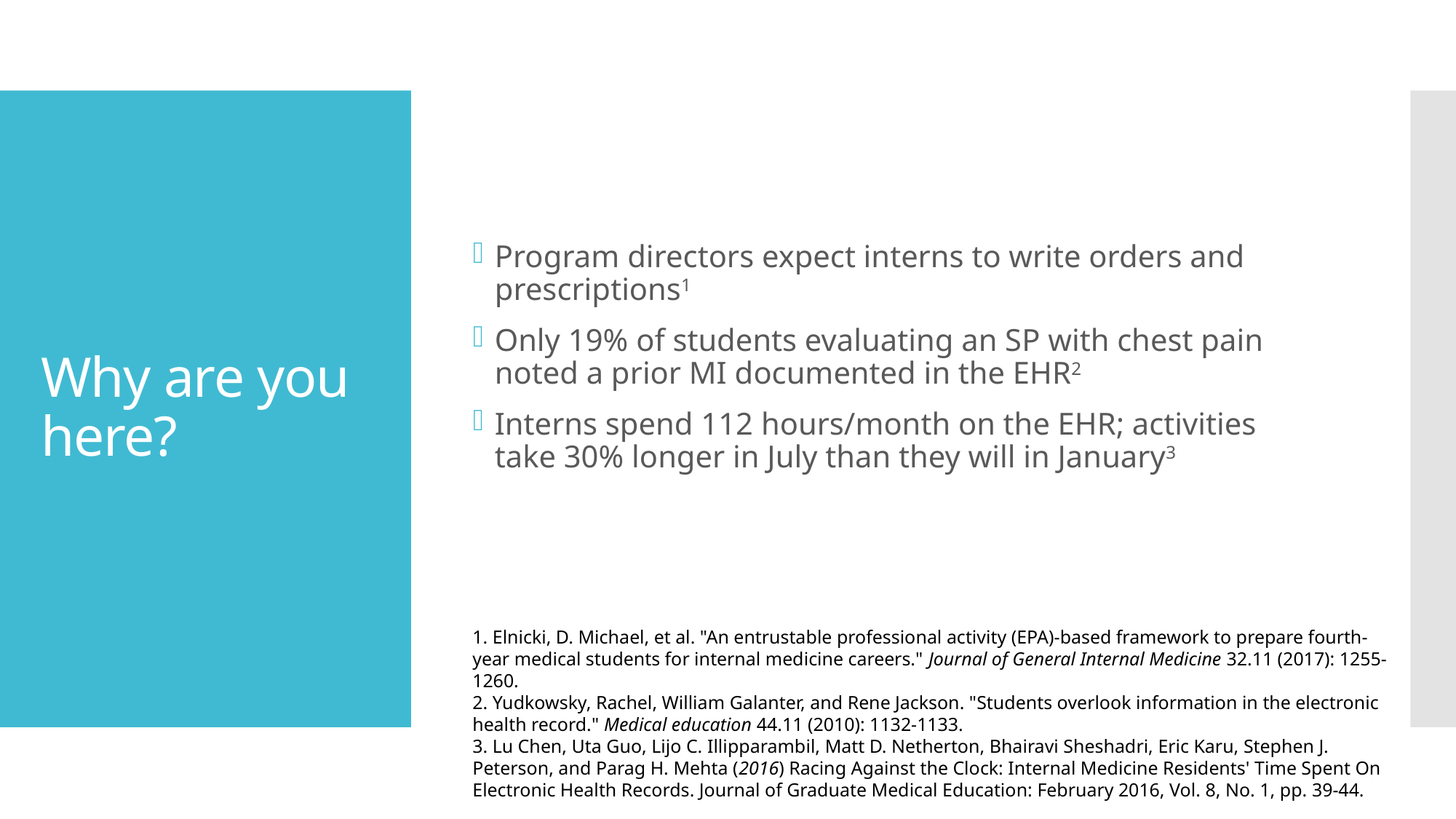

Program directors expect interns to write orders and prescriptions1
Only 19% of students evaluating an SP with chest pain noted a prior MI documented in the EHR2
Interns spend 112 hours/month on the EHR; activities take 30% longer in July than they will in January3
# Why are you here?
1. Elnicki, D. Michael, et al. "An entrustable professional activity (EPA)-based framework to prepare fourth-year medical students for internal medicine careers." Journal of General Internal Medicine 32.11 (2017): 1255-1260.
2. Yudkowsky, Rachel, William Galanter, and Rene Jackson. "Students overlook information in the electronic health record." Medical education 44.11 (2010): 1132-1133.
3. Lu Chen, Uta Guo, Lijo C. Illipparambil, Matt D. Netherton, Bhairavi Sheshadri, Eric Karu, Stephen J. Peterson, and Parag H. Mehta (2016) Racing Against the Clock: Internal Medicine Residents' Time Spent On Electronic Health Records. Journal of Graduate Medical Education: February 2016, Vol. 8, No. 1, pp. 39-44.

## Slide 4
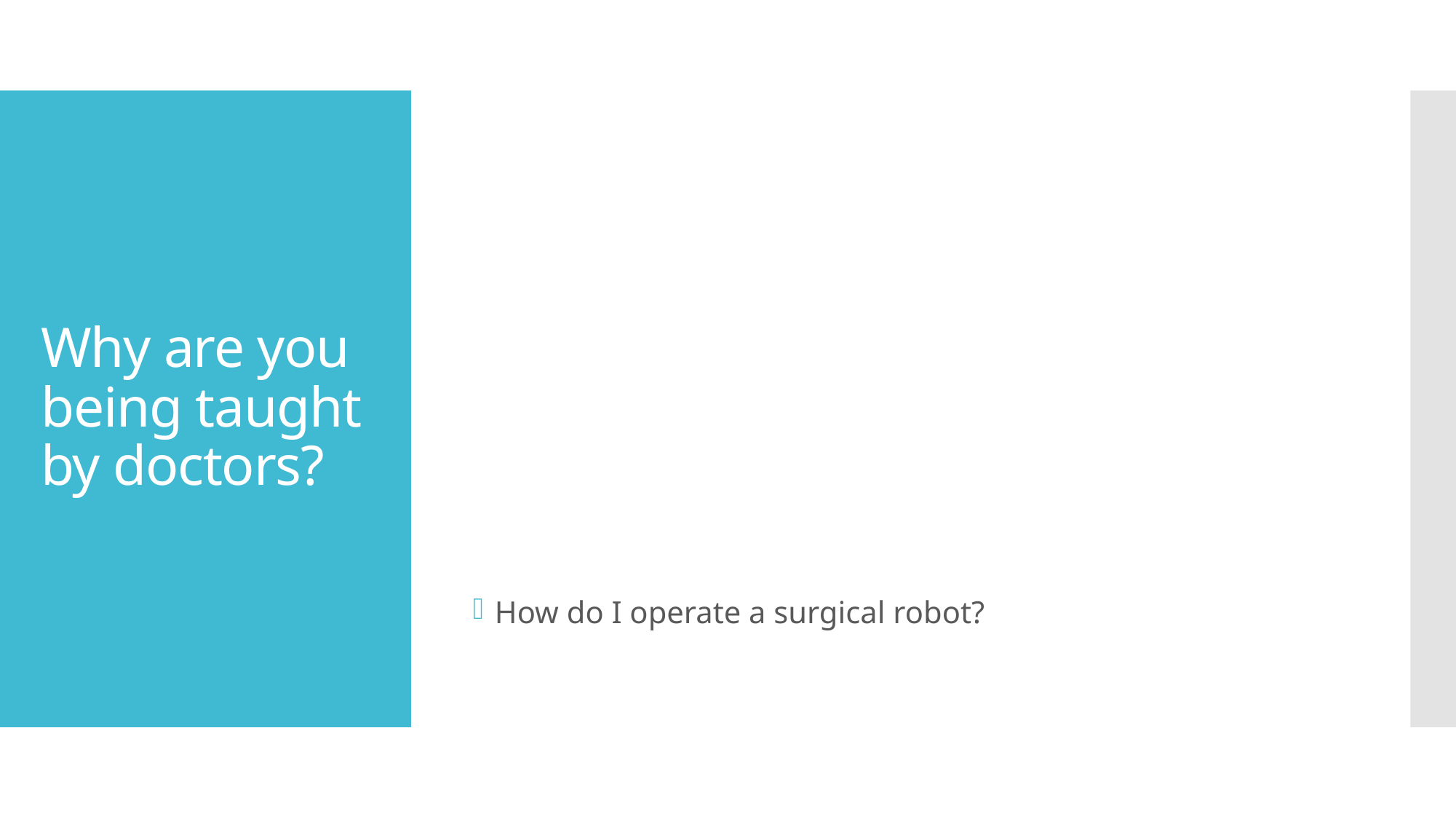

# Why are you being taught by doctors?
How do I operate a surgical robot?

## Slide 5
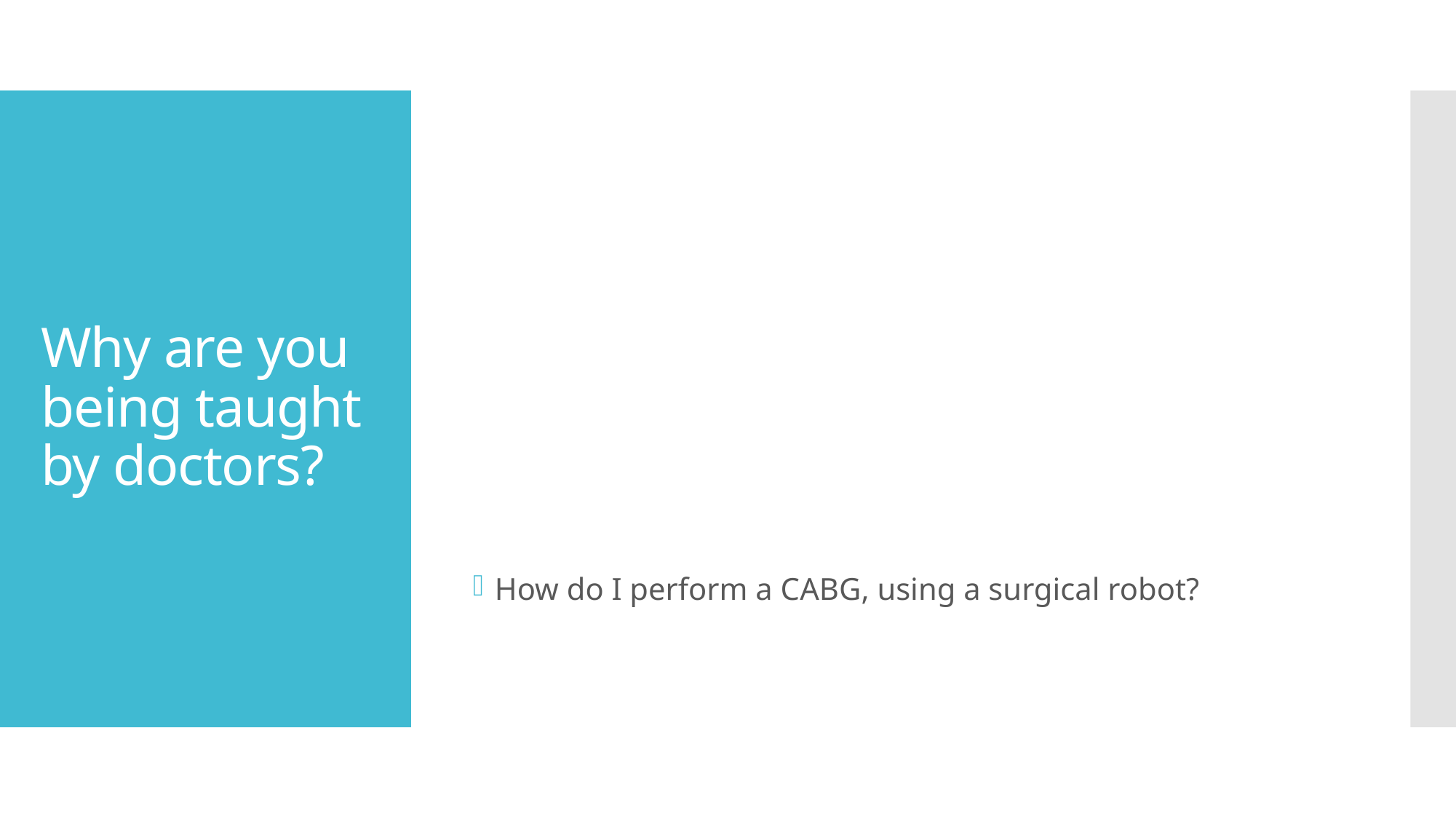

# Why are you being taught by doctors?
How do I perform a CABG, using a surgical robot?

## Slide 6
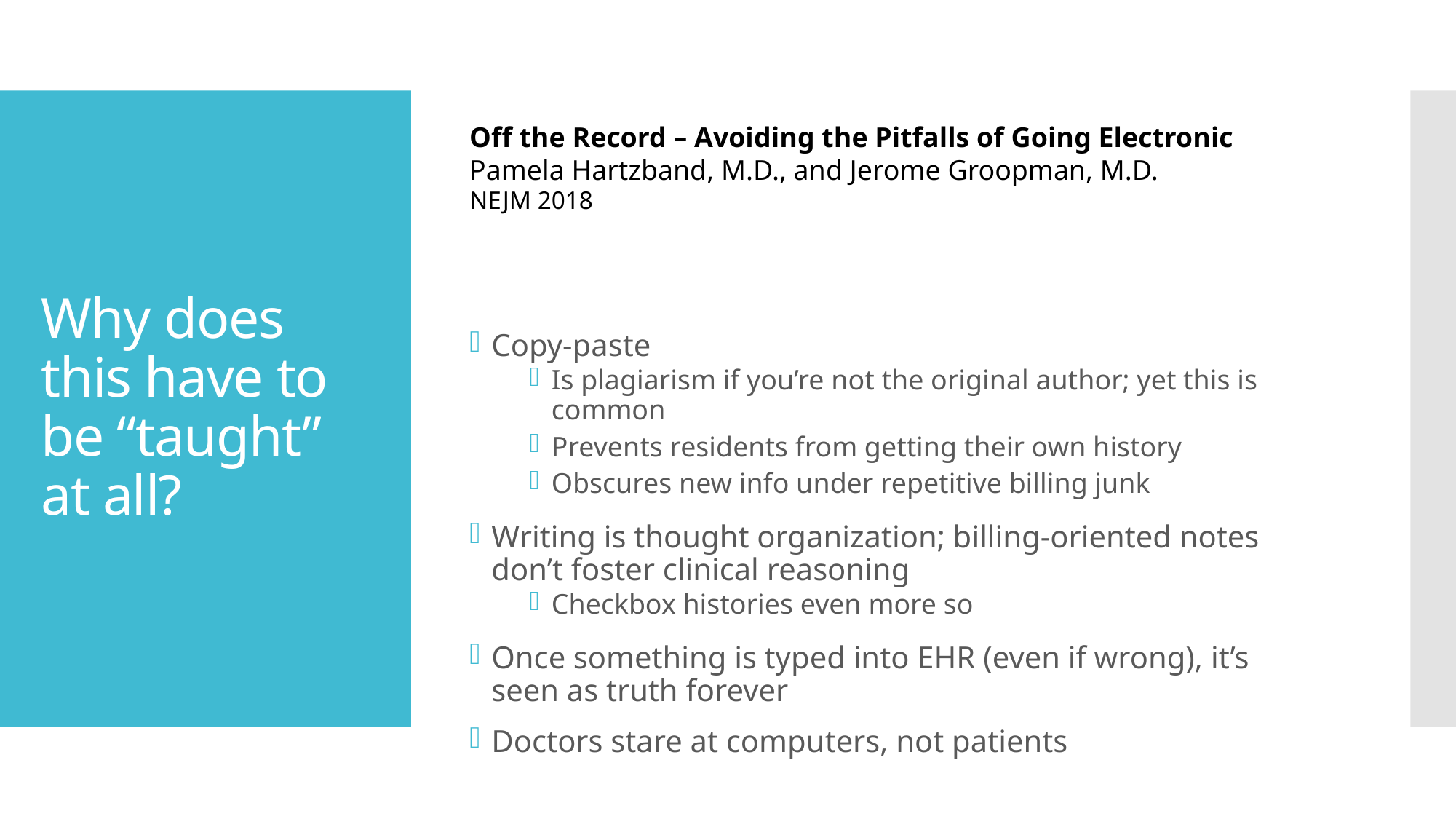

Off the Record – Avoiding the Pitfalls of Going Electronic
Pamela Hartzband, M.D., and Jerome Groopman, M.D.
NEJM 2018
# Why does this have to be “taught” at all?
Copy-paste
Is plagiarism if you’re not the original author; yet this is common
Prevents residents from getting their own history
Obscures new info under repetitive billing junk
Writing is thought organization; billing-oriented notes don’t foster clinical reasoning
Checkbox histories even more so
Once something is typed into EHR (even if wrong), it’s seen as truth forever
Doctors stare at computers, not patients

## Slide 7
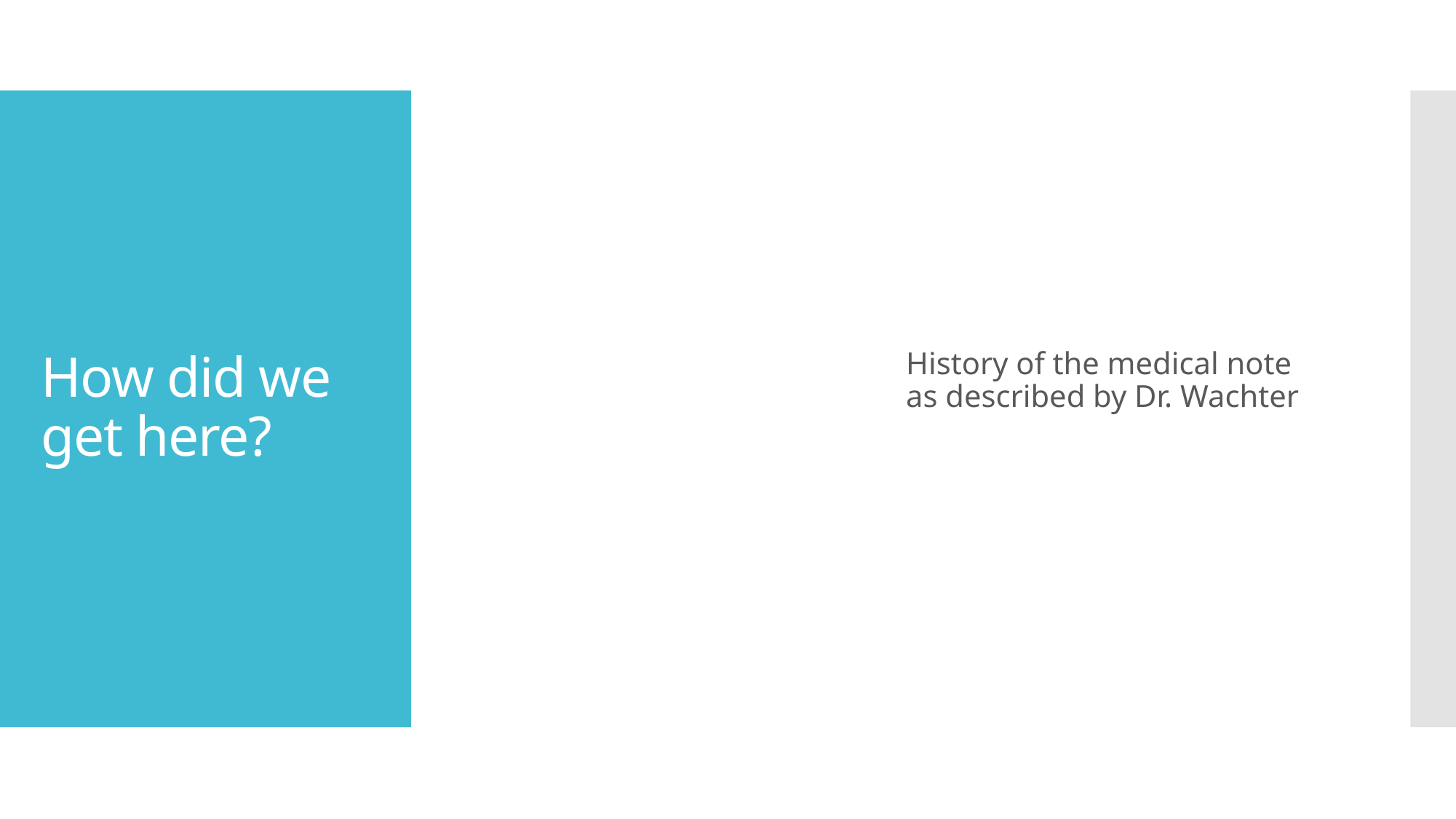

History of the medical note as described by Dr. Wachter
# How did we get here?

## Slide 8
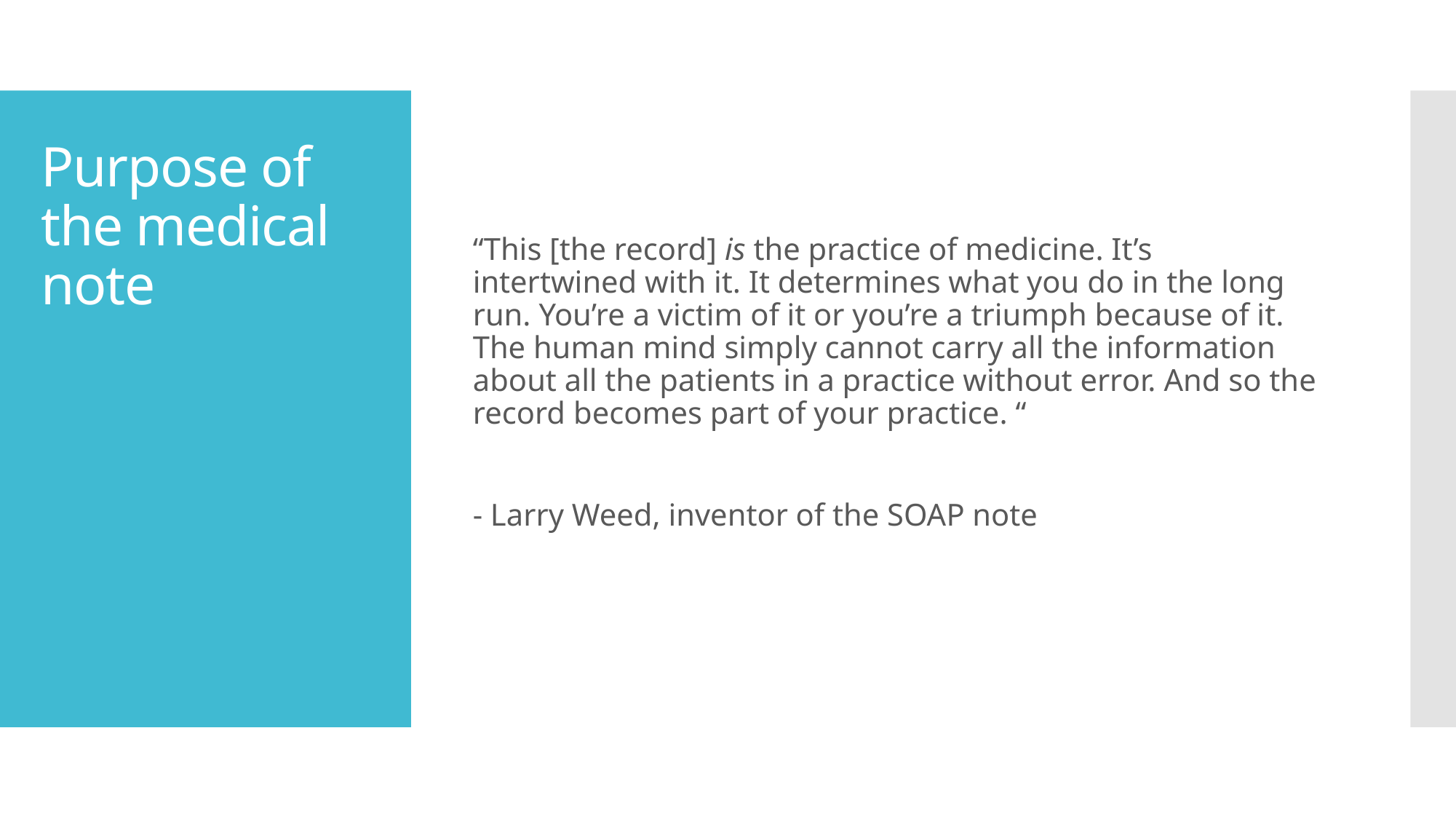

“This [the record] is the practice of medicine. It’s intertwined with it. It determines what you do in the long run. You’re a victim of it or you’re a triumph because of it. The human mind simply cannot carry all the information about all the patients in a practice without error. And so the record becomes part of your practice. “
- Larry Weed, inventor of the SOAP note
# Purpose of the medical note

## Slide 9
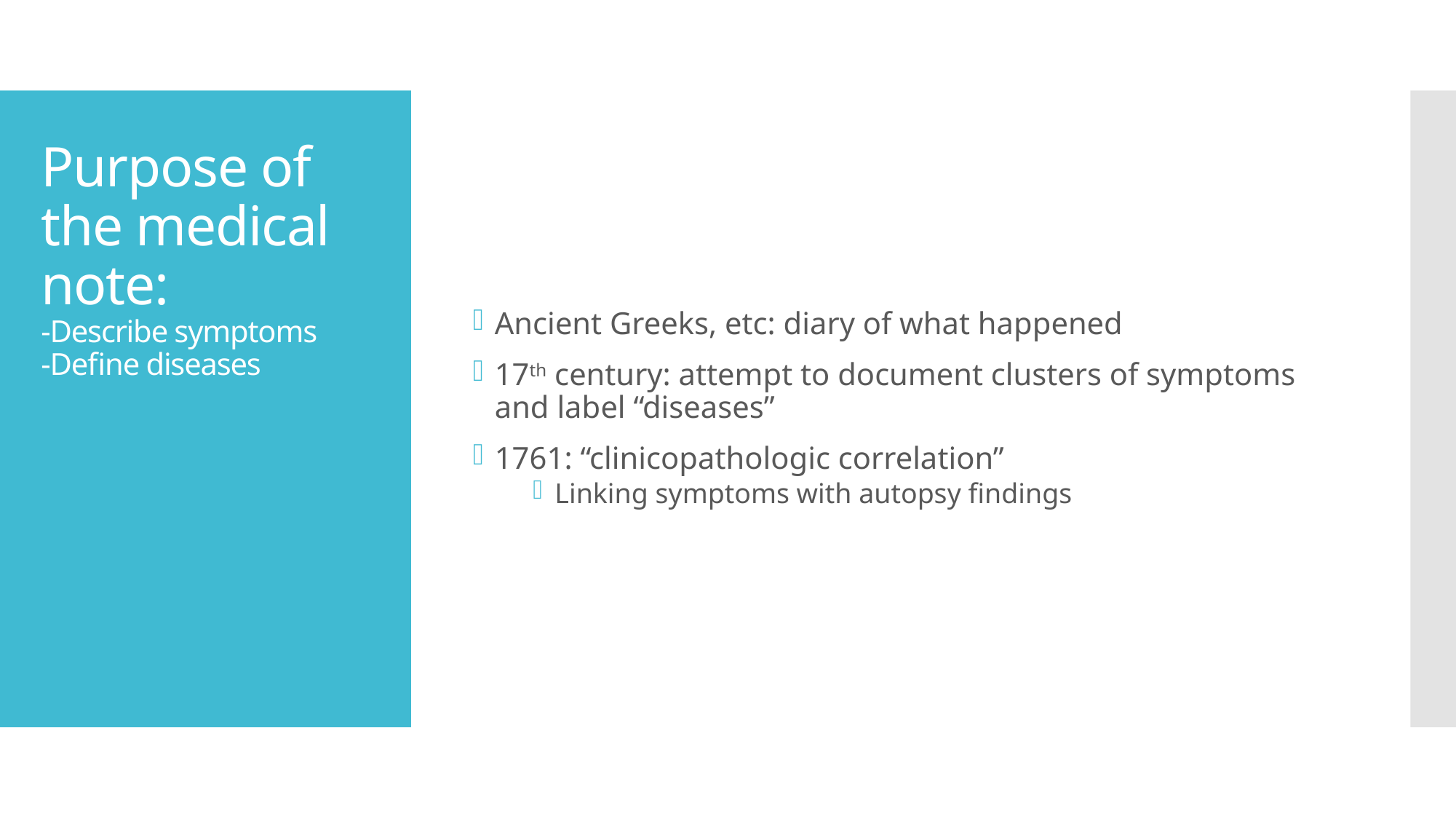

Ancient Greeks, etc: diary of what happened
17th century: attempt to document clusters of symptoms and label “diseases”
1761: “clinicopathologic correlation”
Linking symptoms with autopsy findings
# Purpose of the medical note: -Describe symptoms -Define diseases

## Slide 10
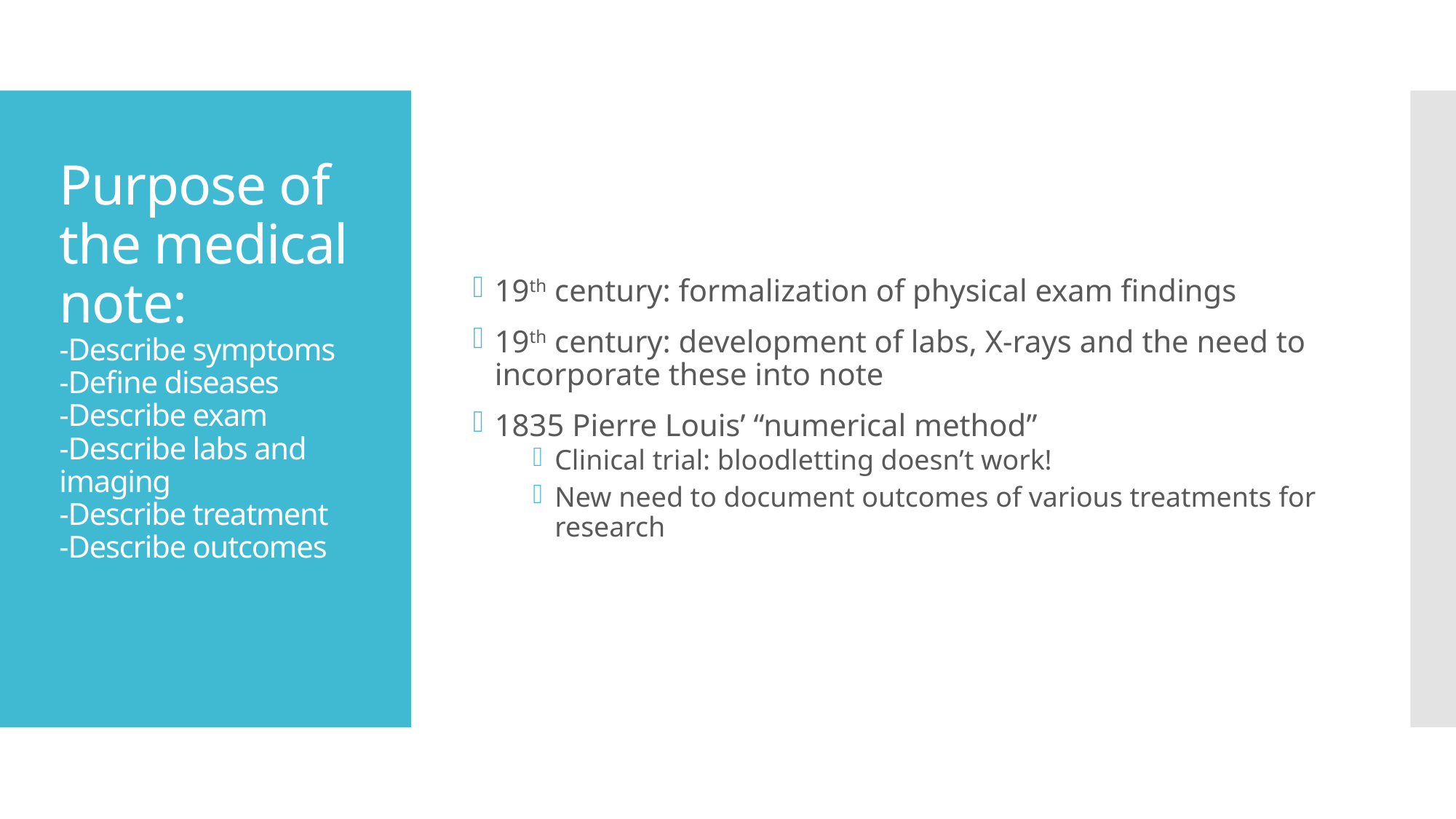

19th century: formalization of physical exam findings
19th century: development of labs, X-rays and the need to incorporate these into note
1835 Pierre Louis’ “numerical method”
Clinical trial: bloodletting doesn’t work!
New need to document outcomes of various treatments for research
Purpose of the medical note:
-Describe symptoms
-Define diseases
-Describe exam
-Describe labs and imaging
-Describe treatment
-Describe outcomes

## Slide 11
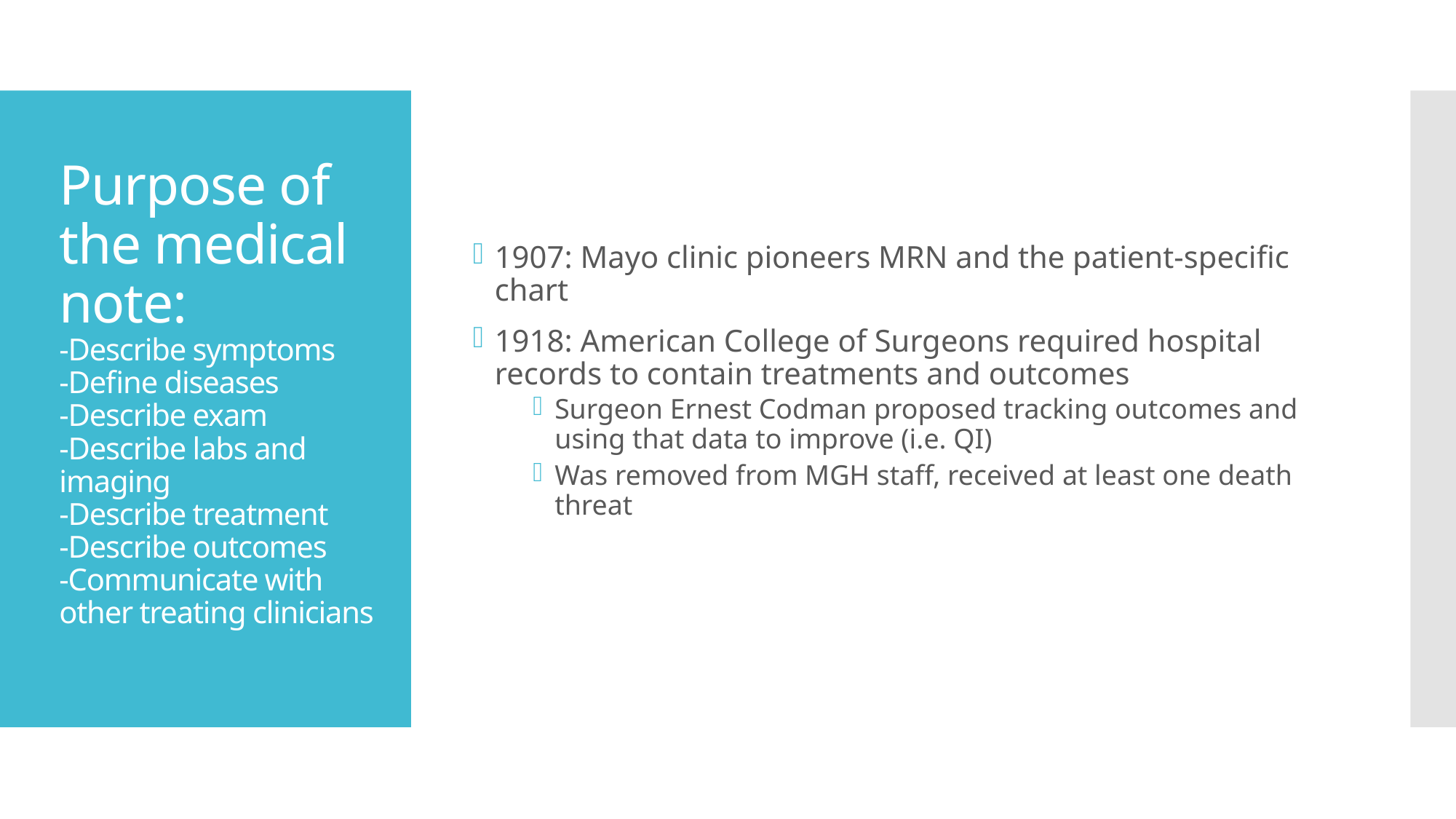

1907: Mayo clinic pioneers MRN and the patient-specific chart
1918: American College of Surgeons required hospital records to contain treatments and outcomes
Surgeon Ernest Codman proposed tracking outcomes and using that data to improve (i.e. QI)
Was removed from MGH staff, received at least one death threat
Purpose of the medical note:
-Describe symptoms
-Define diseases
-Describe exam
-Describe labs and imaging
-Describe treatment
-Describe outcomes
-Communicate with other treating clinicians

## Slide 12
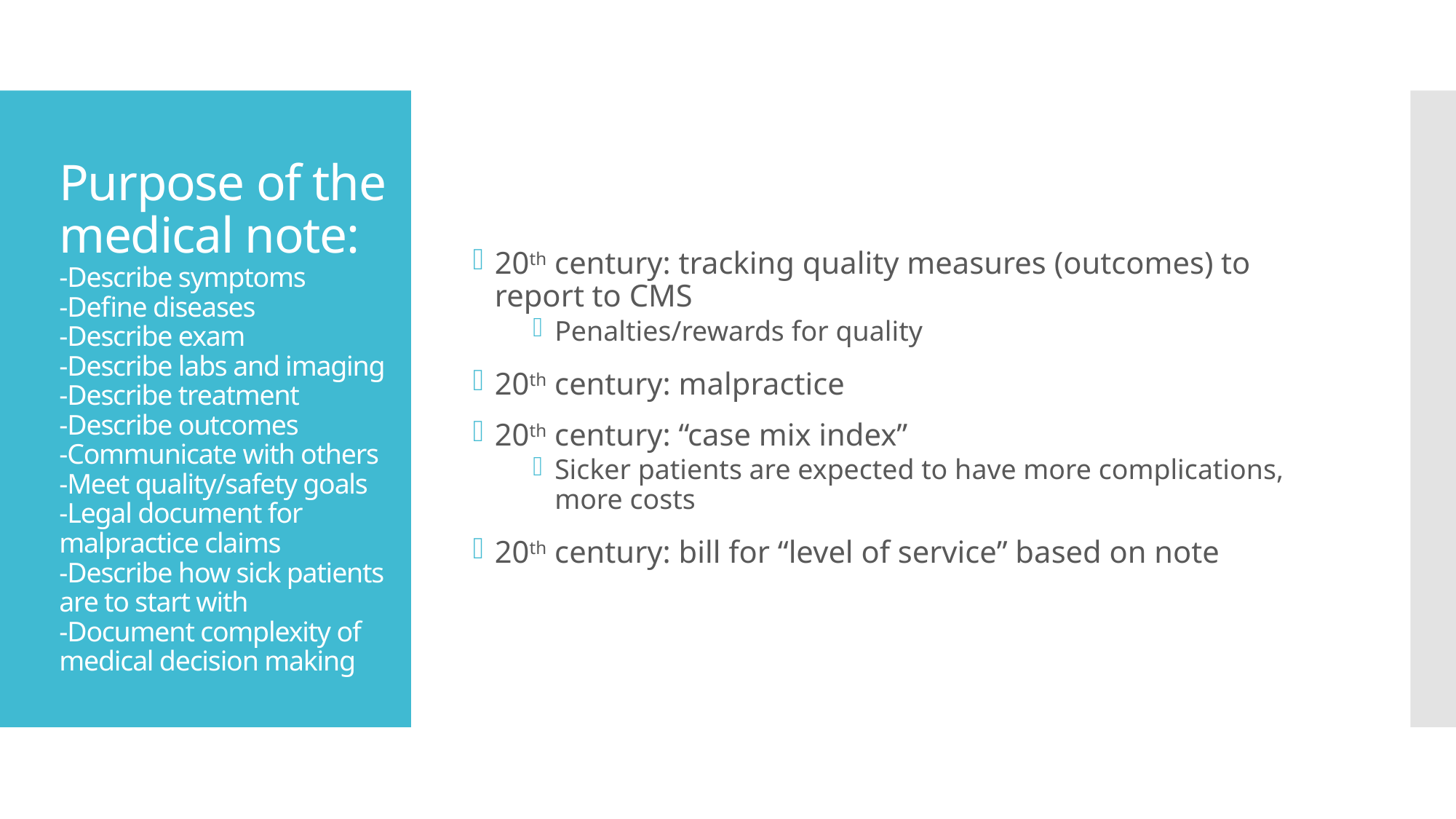

20th century: tracking quality measures (outcomes) to report to CMS
Penalties/rewards for quality
20th century: malpractice
20th century: “case mix index”
Sicker patients are expected to have more complications, more costs
20th century: bill for “level of service” based on note
Purpose of the medical note:
-Describe symptoms
-Define diseases
-Describe exam
-Describe labs and imaging
-Describe treatment
-Describe outcomes
-Communicate with others
-Meet quality/safety goals
-Legal document for malpractice claims
-Describe how sick patients are to start with
-Document complexity of medical decision making

## Slide 13
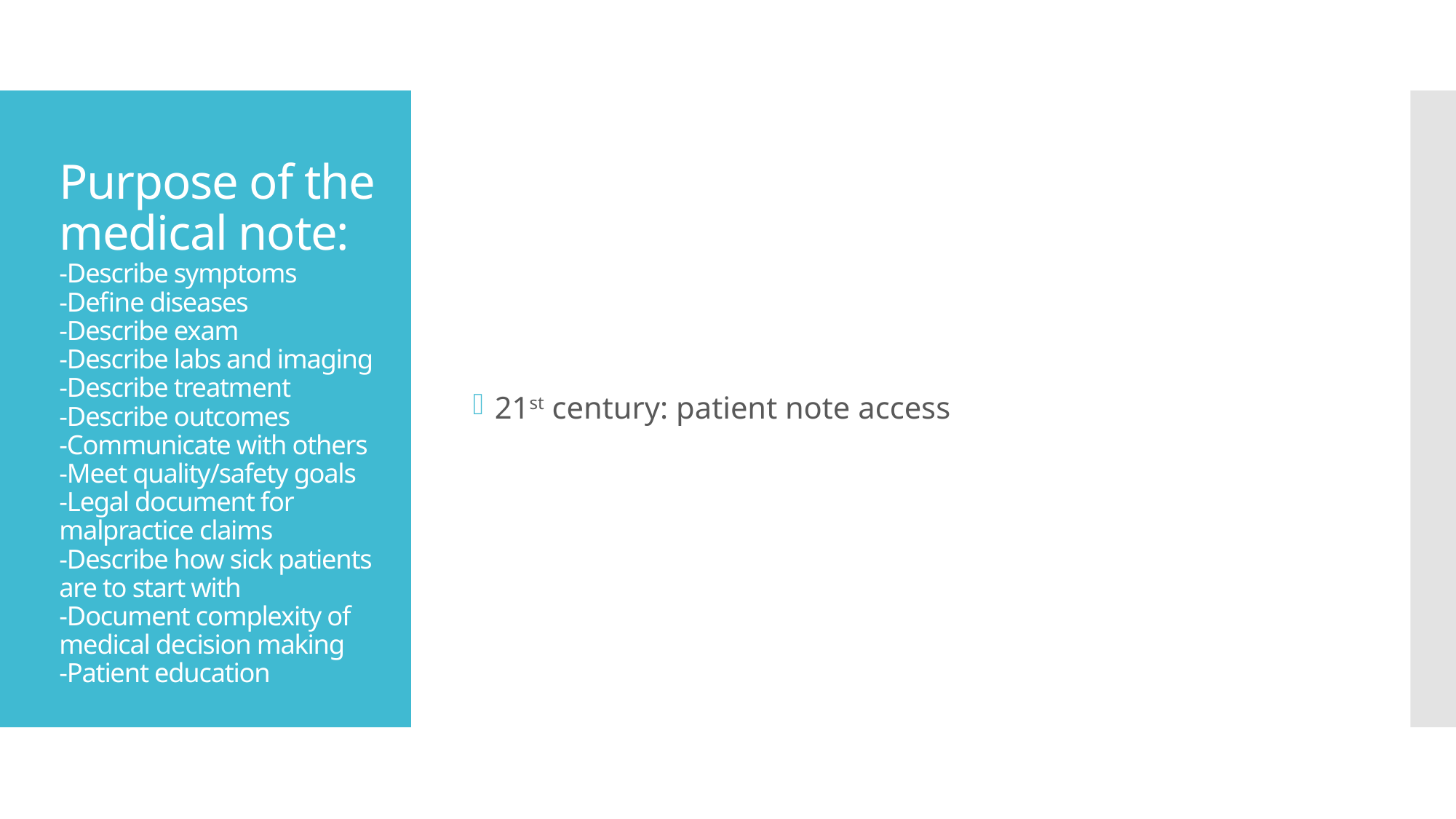

21st century: patient note access
Purpose of the medical note:
-Describe symptoms
-Define diseases
-Describe exam
-Describe labs and imaging
-Describe treatment
-Describe outcomes
-Communicate with others
-Meet quality/safety goals
-Legal document for malpractice claims
-Describe how sick patients are to start with
-Document complexity of medical decision making
-Patient education

## Slide 14
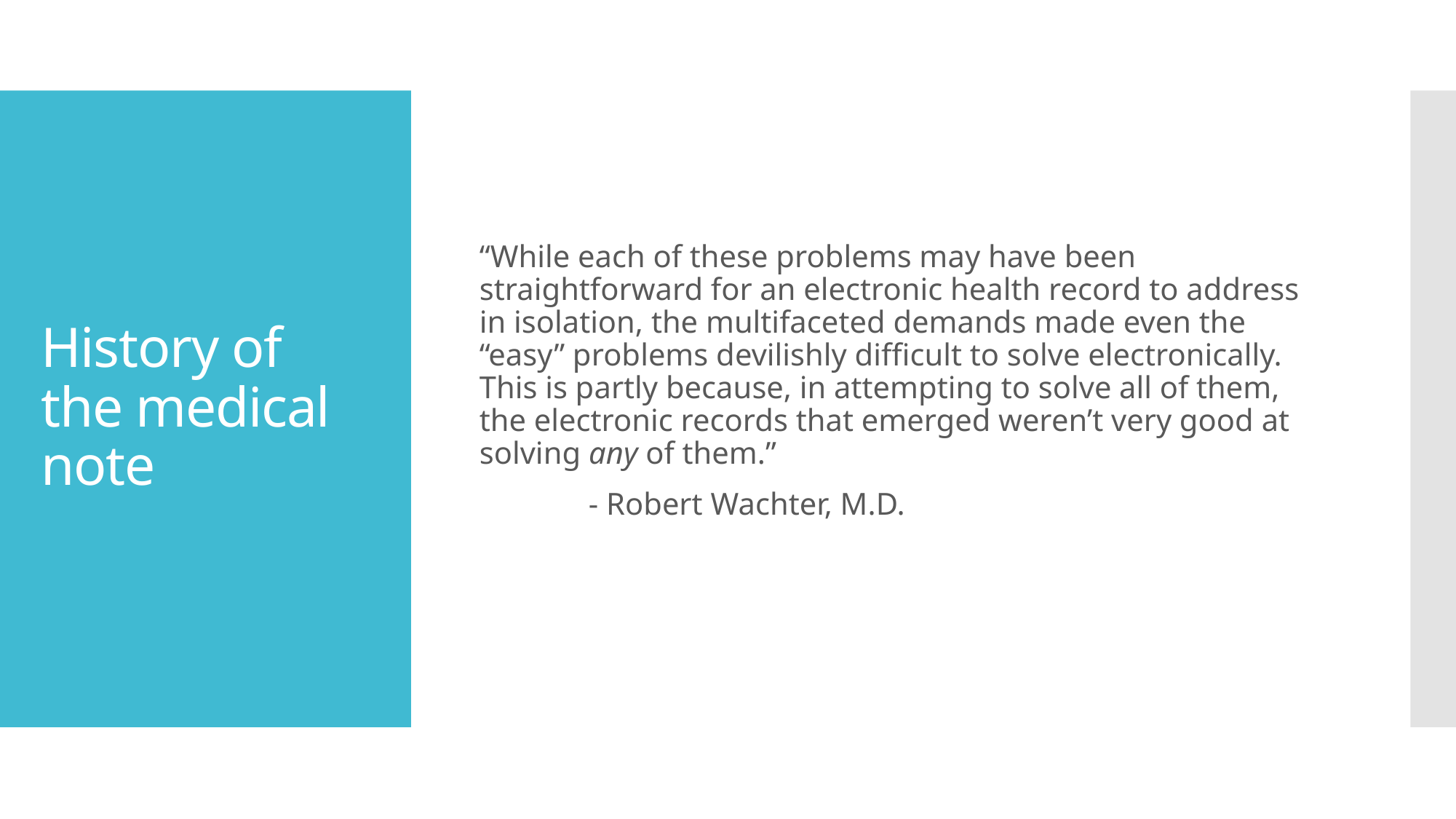

“While each of these problems may have been straightforward for an electronic health record to address in isolation, the multifaceted demands made even the “easy” problems devilishly difficult to solve electronically. This is partly because, in attempting to solve all of them, the electronic records that emerged weren’t very good at solving any of them.”
	- Robert Wachter, M.D.
# History of the medical note

## Slide 15
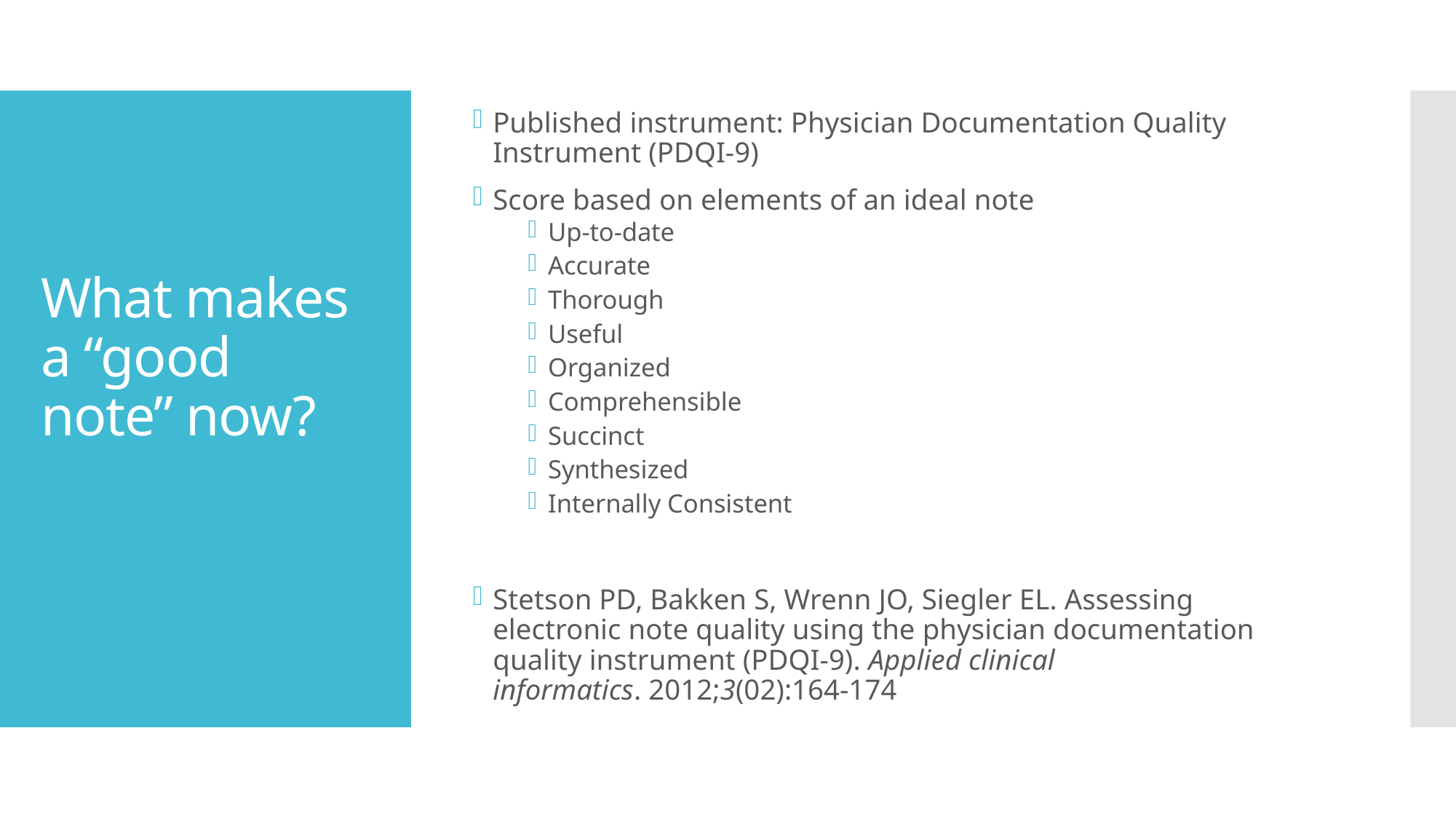

Published instrument: Physician Documentation Quality Instrument (PDQI-9)
Score based on elements of an ideal note
Up-to-date
Accurate
Thorough
Useful
Organized
Comprehensible
Succinct
Synthesized
Internally Consistent
Stetson PD, Bakken S, Wrenn JO, Siegler EL. Assessing electronic note quality using the physician documentation quality instrument (PDQI-9). Applied clinical informatics. 2012;3(02):164-174
# What makes a “good note” now?

## Slide 16
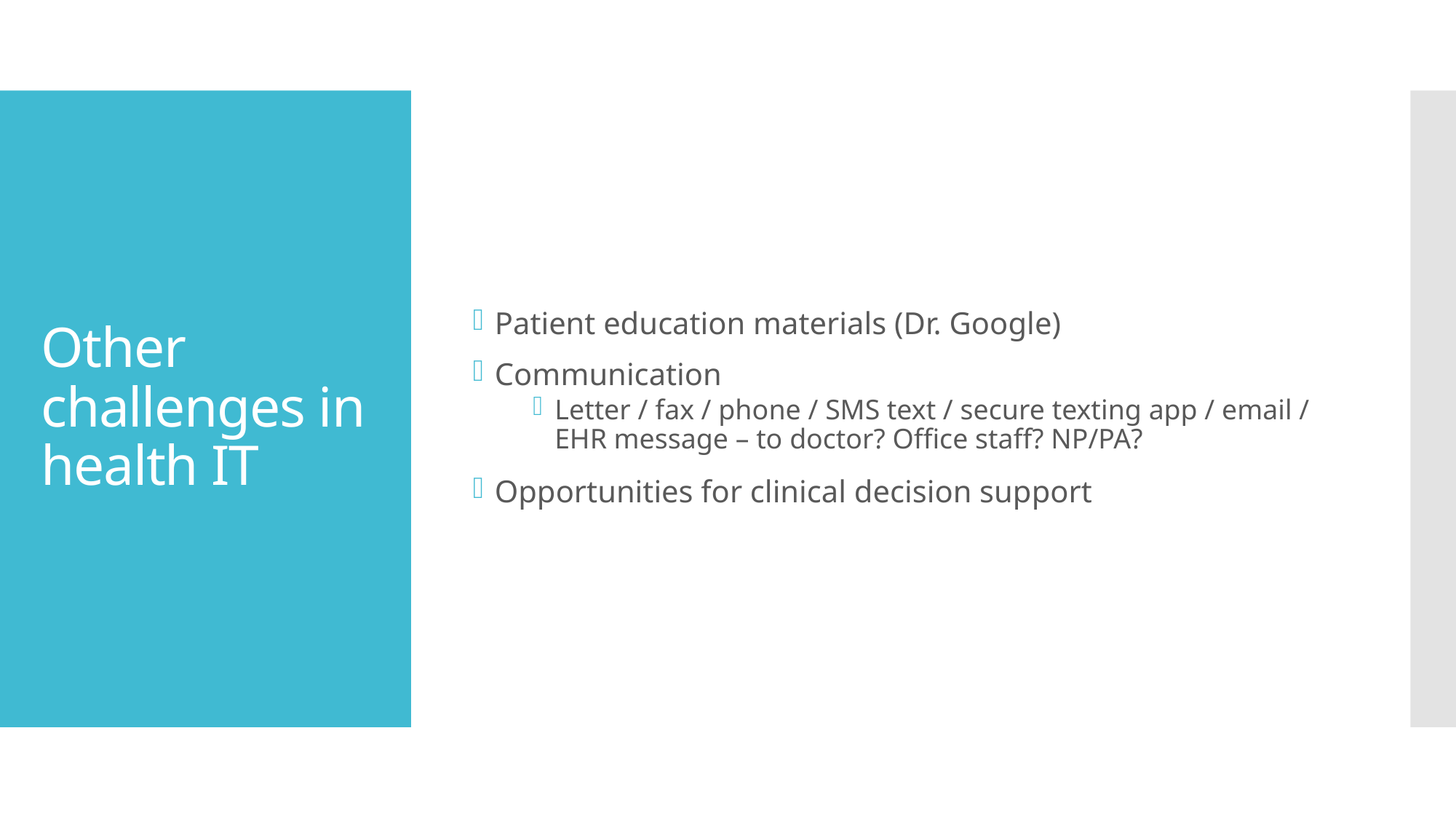

Patient education materials (Dr. Google)
Communication
Letter / fax / phone / SMS text / secure texting app / email / EHR message – to doctor? Office staff? NP/PA?
Opportunities for clinical decision support
# Other challenges in health IT

## Slide 17
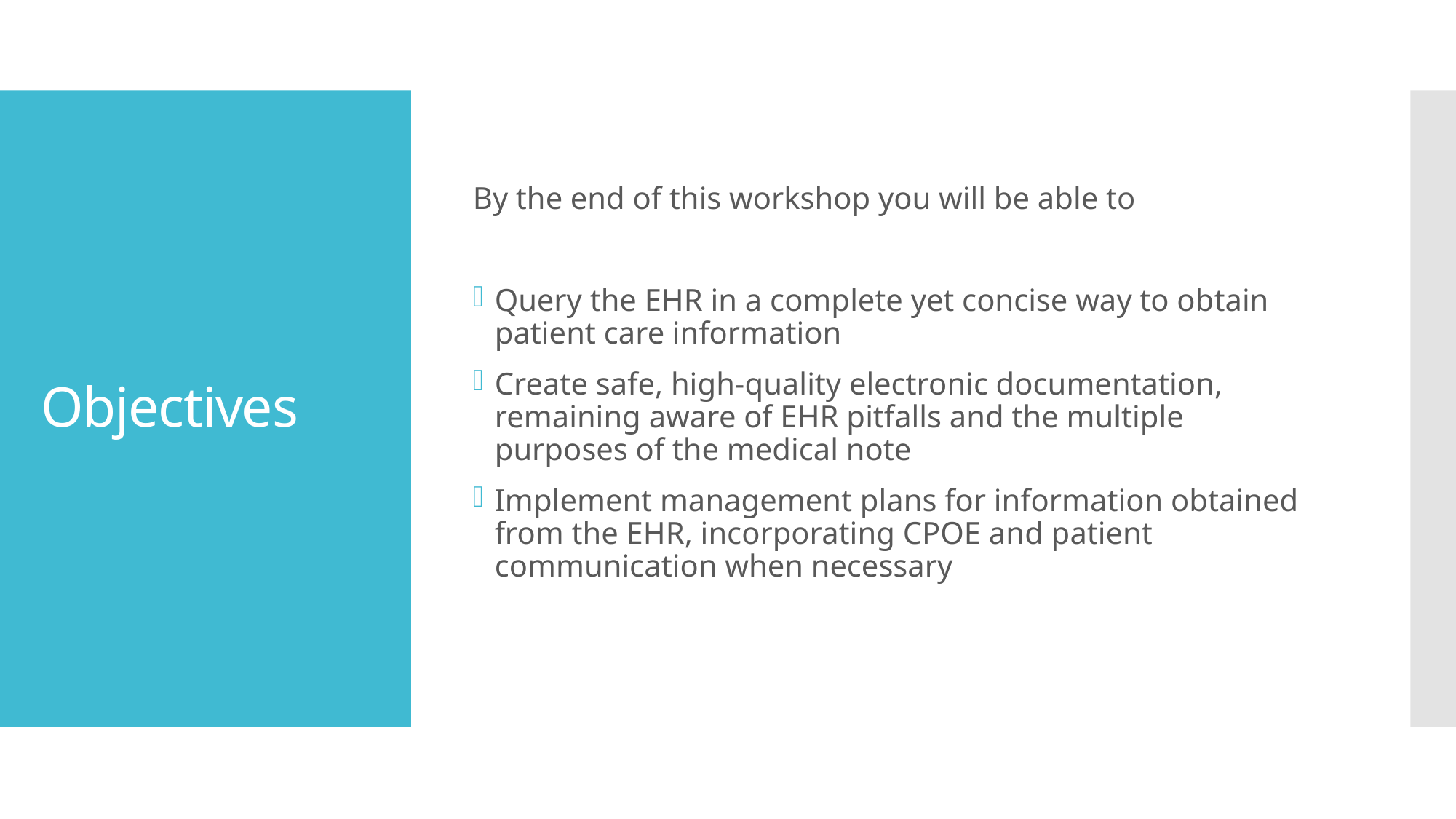

By the end of this workshop you will be able to
Query the EHR in a complete yet concise way to obtain patient care information
Create safe, high-quality electronic documentation, remaining aware of EHR pitfalls and the multiple purposes of the medical note
Implement management plans for information obtained from the EHR, incorporating CPOE and patient communication when necessary
# Objectives

## Slide 18
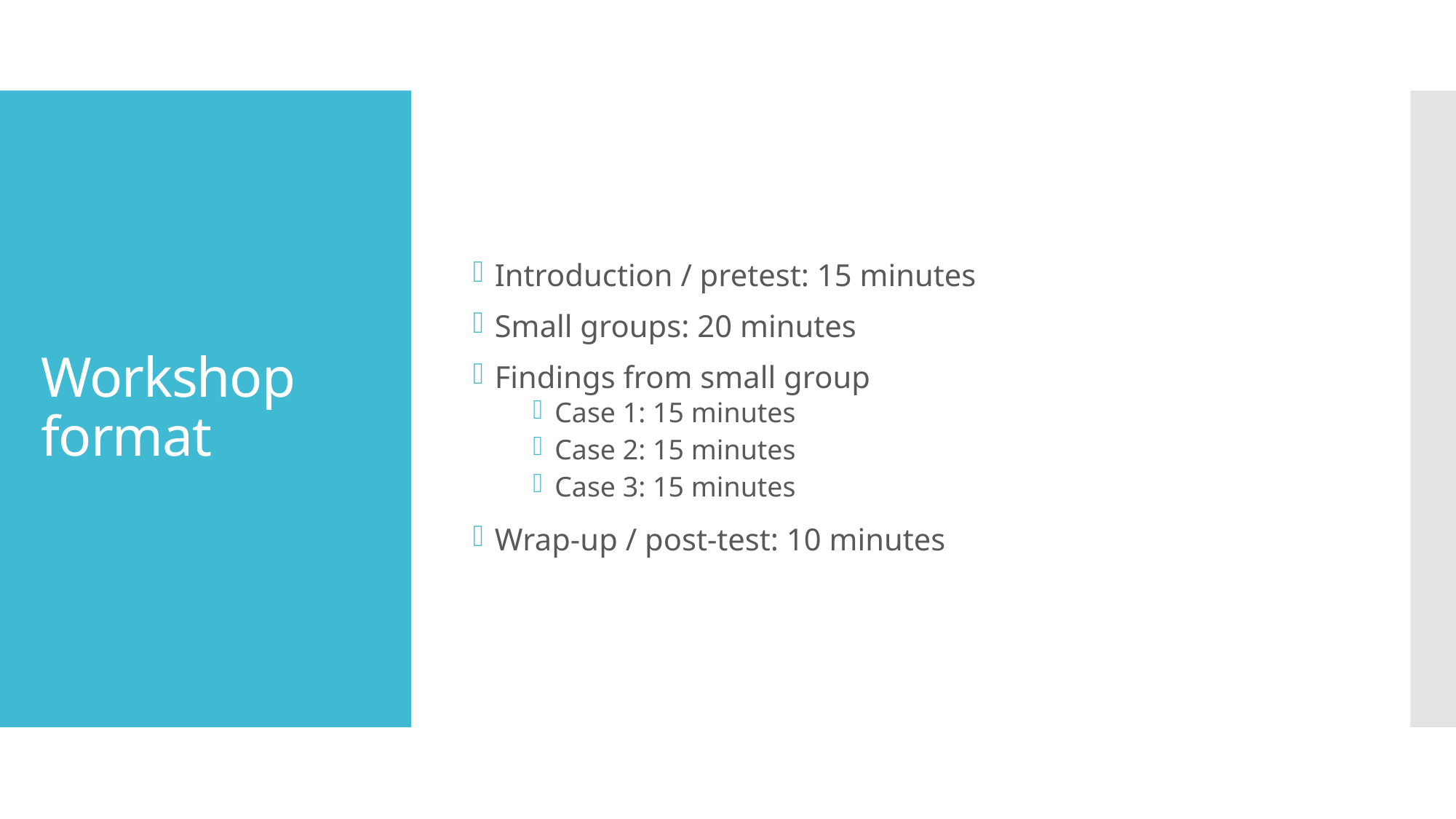

Introduction / pretest: 15 minutes
Small groups: 20 minutes
Findings from small group
Case 1: 15 minutes
Case 2: 15 minutes
Case 3: 15 minutes
Wrap-up / post-test: 10 minutes
# Workshop format
