# Supplementary material for: Electronic Health Record Skills Workshop for Medical Students
Source: MedEdPORTAL. 2019 Oct 25;15:10849. doi: 10.15766/mep_2374-8265.10849 (PMC6946580; doi:10.15766/mep_2374-8265.10849)
Supplement: Supplementary file 1 — A. Case 1.docx B. Case 2.docx C. Case 3.docx D. Student Guide.docx E. Facilitator Guide.docx F. Pretest and Posttest.docx G. EHR Presentation.pptx H. PDQI-9.pdf [file mep-15-10849-s001.zip › H. PDQI-9.pdf]

Appendix: Physician Documentation Quality Instrument (PDQI-9)

Date: __________ **Author:** ___________ **Reviewer:** ______________

Note Type (circle): Admit Progress Discharge

Instructions: Please review the chart before assessing the note. Then rate the note on each of the following attributes:

Attribute	Score	Description of Ideal Note
**1. Up-to-date**	Not at all 1 2 3 4 5 Extremely	The note contains the most recent test results and recommendations.
**2. Accurate**	Not at all 1 2 3 4 5 Extremely	The note is true. It is free of incorrect information.
**3. Thorough**	Not at all 1 2 3 4 5 Extremely	The note is complete and documents all of the issues of importance to the patient.
**4. Useful**	Not at all 1 2 3 4 5 Extremely	The note is extremely relevant, providing valuable information and/or analysis.
**5. Organized**	Not at all 1 2 3 4 5 Extremely	The note is well-formed and structured in a way that helps the reader understand the patient's clinical course.
**6. Comprehensible**	Not at all 1 2 3 4 5 Extremely	The note is clear, without ambiguity or sections that are difficult to understand.
**7. Succinct**	Not at all 1 2 3 4 5 Extremely	The note is brief, to the point, and without redundancy.
**8. Synthesized**	Not at all 1 2 3 4 5 Extremely	The note reflects the author's understanding of the patient's status and ability to develop a plan of care.
**9. Internally Consistent**	Not at all 1 2 3 4 5 Extremely	No part of the note ignores or contradicts any other part.
**Total Score:**		

(Version 1: 11/21/2011)
